# Modulators of the Hop-HSP90 Protein–Protein Interaction Disrupt KSHV Lytic Replication

**DOI:** 10.1021/acsinfecdis.4c00429

**Published:** 2024-10-30

**Authors:** Michael O. Okpara, Michaelone C. Vaaltyn, Jessica L. Watson, Mahama Alhassan, Fernando Albericio, Beatriz G. de la Torre, David J. Clarke, Clinton G. L. Veale, Adrienne L. Edkins

**Affiliations:** † Biomedical Biotechnology Research Unit (BioBRU), Department of Biochemistry and Microbiology, Rhodes University, Makhanda 6139, South Africa; ‡ School of Chemistry and Physics, University of Kwa-Zulu Natal, Durban, Westville 4001, South Africa; § School of Laboratory Medicine and Medical Sciences, University of Kwa-Zulu Natal, Durban 4041, South Africa; ∥ EaStCHEM, School of Chemistry, University of Edinburgh, Joseph Black Building, David Brewster Road, Edinburgh EH93FJ, United Kingdom; ⊥ Department of Chemistry, University of Cape Town, Rondebosch, Cape Town 7701, South Africa

**Keywords:** protein−protein interaction, host-based antiviral targets, KSHV, Hop-HSP90, viral lytic replication

## Abstract

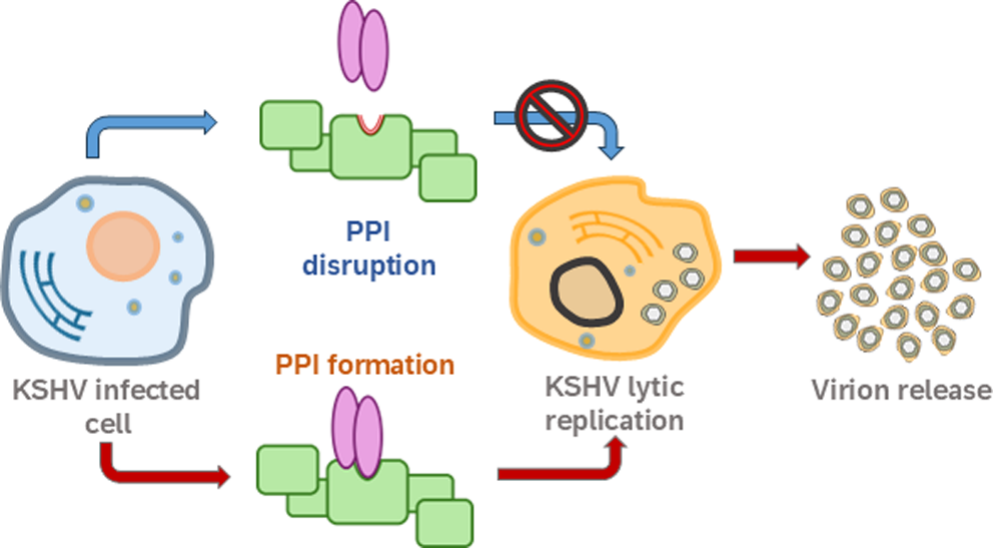

The central role of the chaperome in maintaining cellular proteostasis has seen numerous viral families evolve to parasitically exploit host chaperones in their life cycle. The HSP90 chaperone protein and its cochaperone Hop have both individually been shown to be essential factors for Kaposi sarcoma-associated herpesvirus (KSHV) lytic replication. Given the fundamental regulatory role that protein–protein interactions (PPIs) play in cellular biology, we reasoned that disrupting the Hop-HSP90 PPI may provide a new host-based target for inhibiting KSHV lytic replication. This study expands upon a previous report of non-natural peptides, which were found to disrupt the association between the Hop_TPR2A_ domain and its interacting HSP90_CTD_. Here, in addition to providing insight into the structure–activity relationships of PPI inhibition, we show disruption of the full-length Hop-HSP90 PPI. The inhibitory peptides selectively engaged the Hop_TPR2A_ domain in cell lysates and when tethered to a cell-penetrating peptide acted as noncytotoxic inhibitors of KSHV lytic replication by lowering the viral load, preventing the production of infectious virions, and reducing the expression of KSHV lytic genes. In addition to tentative evidence of Hop-HSP90 PPI as a much-needed target for KSHV drug discovery, this study represents an important step in understanding viral interactions with the host proteostasis machinery.

Molecular chaperones regulate a plethora of intra- and extracellular functions, including protein folding, proteasomal turnover, and translocation, and are essential for cellular proteostasis and cell survival.^1^ The evolution of ever more complex protein folds precipitated the evolution of nonclient cochaperones, which work in concert with core chaperones to maintain a well-functioning proteome.^2,3^ This essential function extends to viruses, which parasitize the host chaperome for viral protein processing and, ultimately, progression through their infection cycle.^4−6^ This is particularly pronounced with mammalian HSP90, an abundant and evolutionary conserved chaperone upon which numerous DNA and RNA viruses at various stages of their infection cycle depend.^7,8^ Alongside its potential as a target for cancer drug discovery, inhibition of HSP90 function is considered a promising strategy for inhibiting viral replication.^9,10^ In addition, drug resistance is much less likely to emerge from targeting a host-based factor in the viral life cycle, while at the same time, the likelihood of pan-viral activity is also enhanced.^11^

Kaposi sarcoma-associated herpesvirus (KSHV; or human herpesvirus 8/HHV8) is one of 7 known human oncoviruses contributing to ∼15% of cancers worldwide.^12^ KSHV is required for the development of all forms of Kaposi’s sarcoma (KS), a highly vascular tumor of endothelial lymphatic origin, which is an epidemic AIDS-defining cancer in sub-Saharan Africa.^13,14^ While the introduction of antiretroviral therapy (ART) has led to reductions in KS incidence, it is still disproportionally prominent in Africa,^13^ and KS-associated immune reconstitution inflammatory syndrome (KS-IRIS) is a significant contributor to KS-related mortality.^15^ Despite this, there are currently no targeted therapies for KS or the causative KSHV, with the current standard of care relying on a combination of immunotherapy and nontargeted chemotherapeutics.^16^

Through the dormant latent phase of KSHV infection, the viral genome exists symbiotically with the host genome as extrachromosomal episomes. During this time, no infectious virions are produced, and only a few latent viral genes are expressed.^17^ This property circumvents the host immune system and permits persistent infection. However, disruption of viral latency triggers lytic replication in a three-phase expression cascade (immediate early, delayed early, and late lytic phases), in which the entire viral genome is expressed.^18^ The lytic phase initiates the production of infectious virions disseminating the virus to new sites, including new hosts.^18,19^ Accordingly, KSHV lytic replication is essential for viral propagation and is central to KSHV pathogenicity and KS tumorigenicity.^20^

Commencing with KSHV ORF50, lytic genes associated with the immediate early phase are produced promptly after reactivation. Replication and transcription activator (RTA), translated from KSHV ORF50, activates KSHV lytic promoters and, thus, the switch from latent to lytic infection. Proteins emanating from immediate early genes go on to induce transcription in delayed early genes as well as disrupt host cell processes, to further potentiate KSHV infection.^21^ Therefore, inhibiting KSHV lytic replication, particularly at the earliest possible stages, offers a potential strategy for disrupting the KSHV pathogenesis.

Host HSP90 plays an important role in stabilizing proteins involved in both lytic and latent KSHV infection, including chaperoning numerous late- and early-stage transcription factors where its inhibition is sufficient to either block KSHV replication without generalized cytotoxicity or can selectively induce apoptosis of KSHV-infected cells. Therefore, disruption of this chaperone function is considered a promising strategy for treating KSHV and, by extension, KS.^22−25^ However, inhibition of HSP90, particularly at the *N*-terminal ATPase domain, results in a compensatory heat shock response (HSR), in which heat shock factor 1 (HSF1) induces the upregulation of alternative HSPs, including HSP70, helping to retain cellular proteostasis, thus partially mitigating the effectiveness of HSP90 inhibition.^26,27^

The HSP70–HSP90 organizing protein (Hop) is a cochaperone, which forms sequential transient protein–protein interactions (PPIs) with both HSP70 and HSP90, facilitating the transfer of partially folded client proteins between the two, thus possessing a chaperone mediatory role.^28^ In contrast to noncancerous cells, Hop is constitutively associated with HSP90 in cancer cells^29^ and has been identified as a specific factor associated with invasive or aggressive tumors and tumor metastases.^30−32^ Furthermore, Hop associates with HSP90 when complexed to viral client proteins.^33^ Critically, Hop depletion reduces basal HSF1 levels, cell proliferation, and long-term cell survival.^34^ Accordingly, disruption of the Hop–HSP90 PPI has been postulated as an alternative mode of disrupting chaperone-mediated proteostasis without stimulating the compensatory HSR.^35−37^ Recently, we showed that Hop is required for KSHV lytic replication, with Hop depletion resulting in reduced transcription of KSHV lytic genes, reduced viral DNA load, and reduced infectious virion production.^38^

Several studies have reported the development of peptides whose binding to the HSP90-C-terminal domain (HSP90_CTD_) disrupted PPI formation with Hop, which, in turn, correlated with inhibition of HSP90 activity.^39−41^ Compounds, which interact with the TPR2A domain of Hop (Hop_TPR2A_), have also been explored as an alternative strategy of disrupting the Hop–HSP90 PPI.^42−44^ This included our own reports of a non-natural tetrazole-containing peptide (**1**, Figure 1), which disrupts the Hop_TPR2A_– HSP90_CTD_ domain–domain interaction.^45,46^ Given HSP90s central role in both KSHV lytic replication and KSHV-mediated tumorigenesis, we surmised that disruption of the Hop-HSP90 PPI may be a compelling strategy to inhibit KSHV replication and, by extension, tumor formation. Accordingly, in this study, we build upon our previously reported data to show that peptide **1** alongside a previously unreported non-natural peptide (**2**) is capable of disrupting the full-length Hop-HSP90 PPI. We further demonstrate that by enhancing cell permeability through the addition of an Arg-8 cell-penetrating peptide (CPP), modified versions of peptides **1** and **2** can inhibit KSHV lytic replication, disrupt intracellular HSP90 function, and, for the first time, provide a tentative pharmacological link of the Hop-HSP90 PPI to genotypic and phenotypic alterations in KSHV.

**Figure 1:**
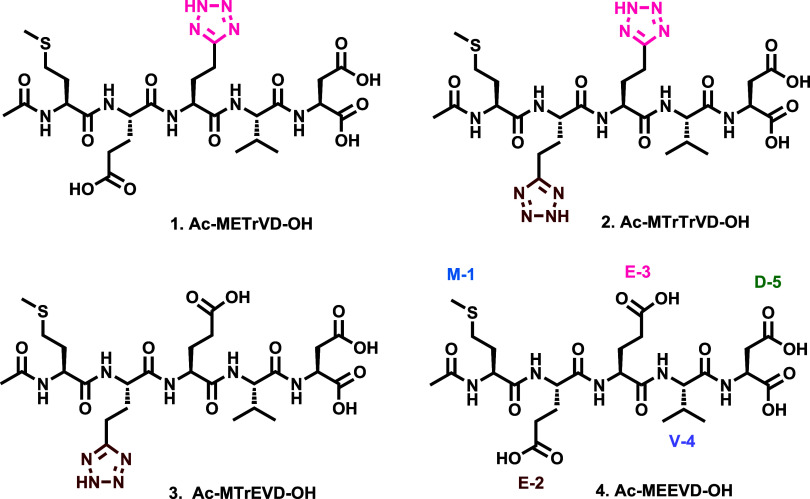
Previously reported peptides **1** and **3** alongside new peptide **2** were rationally designed to mimic peptide **4** and disrupt the Hop_TPR2A_ – HSP90_CTD_ PPI. Peptide **4** represents the native C-terminal HSP90 peptide identified as the minimal binding epitope for PPI formation with Hop_TPR2A_.

## Results and Discussion

### Structure–Activity Relationship of PPI Disruption

The interfacial association between Hop and HSP90 is mediated primarily by an interaction between the “carboxylate clamp” region of Hop_TPR2A_ and an acidic-rich MEEVD pentapeptide motif found at the HSP90_CTD_.^42,47^ This information led to the rational design of peptides **1** and **3** (Figure 1), where one of the central glutamic acid residues of MEEVD (E-2 and E-3) was bioisosterically replaced with a slightly larger, aromatic tetrazole-containing isostere, while retaining (*S*) stereochemistry throughout. Through an ELISA-based PPI solid-phase assay,^48^ we found that both non-natural peptides could disrupt the Hop_TPR2A_–HSP90_CTD_ PPI in a dose-dependent fashion, with peptide **1** possessing the most potent activity. At the same time, an acetylated MEEVD analogue (**4**) was not active. The superior activity of peptide **1** over peptide **2** intimated that the substitution of the E-2 or E-3 glutamic acid residues had a differential impact on PPI stability.^45^

**Figure 2:**
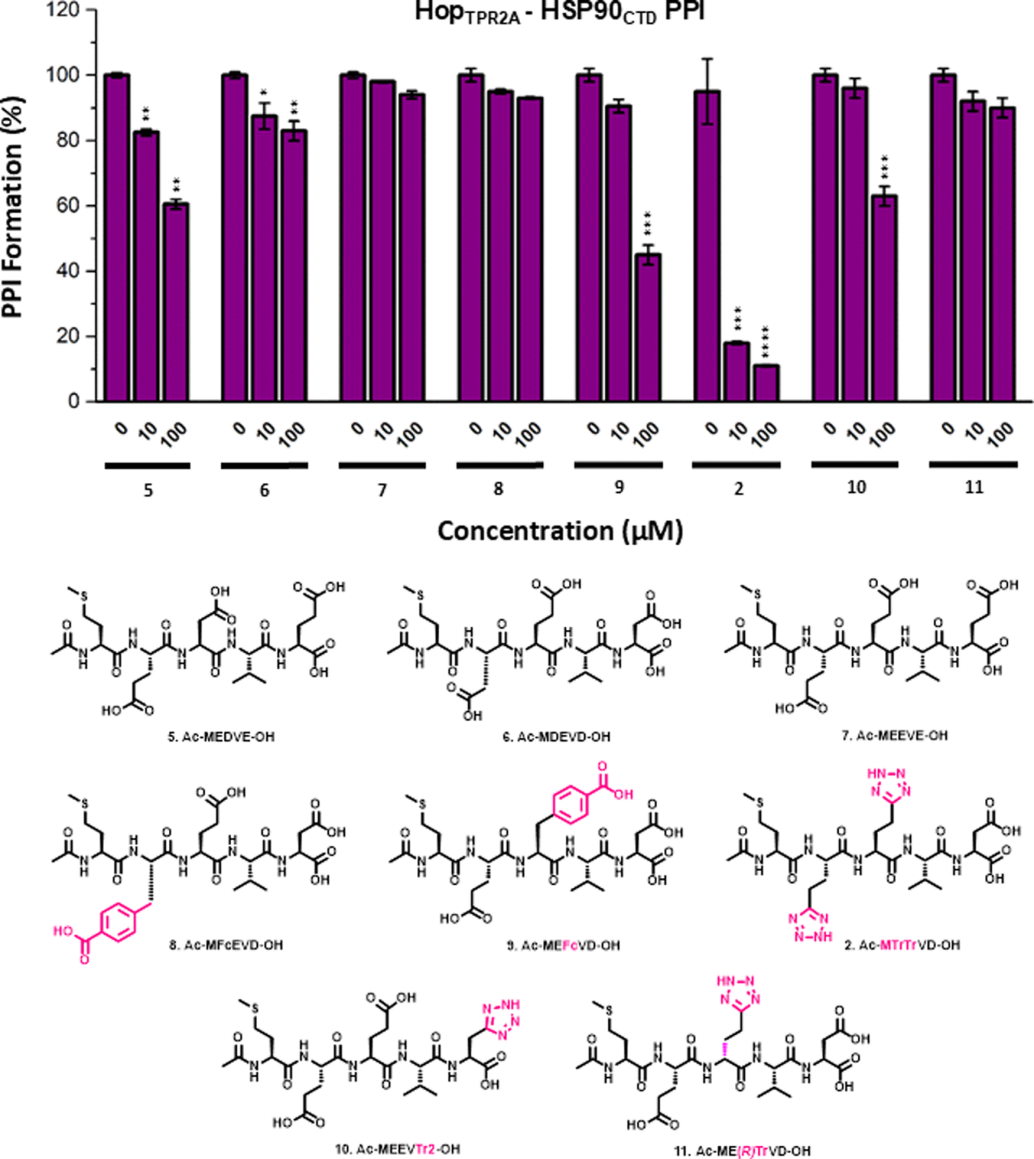
Solid-phase Hop_TPR2A_ – HSP90_CTD_ PPI inhibitory assay (top) in the presence of peptides **2**, **5–11** (bottom). This portion of the SAR focused primarily on the alteration of acidic E and D residues of peptide **4**. Noncanonical amino acid residues are highlighted in pink. Statistical significance was determined by *t* test where *p < 0.01, **p < 0.001, ***p < 0.0005, ****p < 0.0001.

To further probe these insights, we conducted a small structure–activity relationship (SAR, Figure 2). The first cohort of compounds (**2**, **5**–**11**, Figure 2) revolved primarily around exploring the acid-containing D and E residues while conserving the methionine (M-1), which is reportedly important for Hop_TPR2A_ selectivity as well as valine (V-4), which forms a key hydrophobic interaction with Hop_TPR2A_.^49,50^ While translocation of E-3 and D-5 (**5**) resulted in moderate PPI disruption activity, replacement of E-2 with D (**6**) or D-5 with E (**7**) was resulted in a complete loss of activity. Interestingly, while the isosteric replacement of E-2 with 4-carboxyphenyl alanine (**8**) was also inactive, its E-3 substituted isomer (**9**) possessed some moderate activity, suggesting some tolerance for larger aromatic substituents in this area.

Double isosteric replacement of E-2 and E-3 with a tetrazole moiety (**2**) resulted in potent activity analogous to that observed for peptide **1**. However, this pattern did not extend to replacing D-5 with a tetrazole analogue (**10**), which was poorly active. Finally, the E-3 epimer of peptide **2** (**11**) was inactive, highlighting an important stereochemical role in peptide bioactivity.

**Figure 3:**
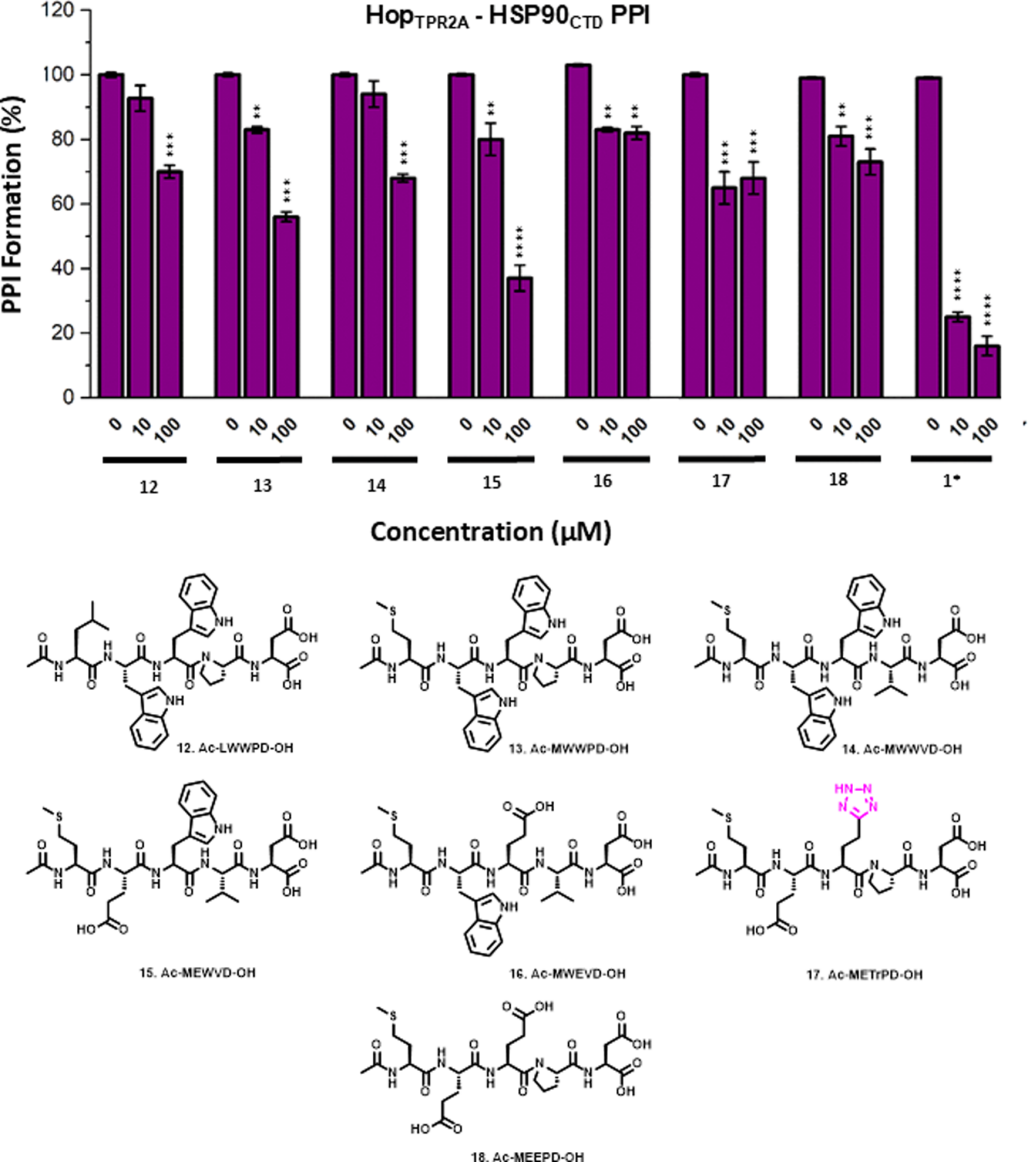
Solid-phase Hop_TPR2A_–HSP90_CTD_ PPI inhibitory assay (top) in the presence of peptides **12–18** (bottom). Peptides were designed to mimic the CHIP interacting with the LWWPD motif. These data, together with that in Figure 2, suggest that major modifications of the MEEVD/METrVD motif are mostly restricted to amino acid 3. Furthermore, alteration of the valine-aspartic acid motif is not tolerated. Noncanonical amino acid residues are highlighted in pink. Peptide **1** was used as a control for all solid-phase Hop_TPR2A_–HSP90_CTD_ PPI inhibitory assays. Statistical significance was determined by *t* test where *p < 0.01, **p < 0.001, ***p < 0.0005 ****p < 0.0001.

The carboxy-terminus of HSP70-interacting protein (CHIP/STUB1) is an E3 ligase, which binds to the C-terminal EEVD motif of both HSP70 and HSP90, leading to ubiquitination and turnover of HSP clients.^51,52^ A study by Gestwicki and co-workers showed that CHIP could interact with a wide range of aspartic acid-terminating pentapeptides with greater affinity than MEEVD. Their positional scanning synthetic library ultimately identified acetylated LWWPD (**12**, Figure 3) as the strongest CHIP-interacting peptide.^53^ We reasoned, therefore, that peptide **12**, in addition to several analogues (**13–18**), which incorporated structural elements from series one peptides, may provide additional SAR insight. Overall, peptides featuring a double E to W substitution (**12**, **13**, and **14**) displayed weak to moderate activity. Some positional preferences for E to W substitution were observed in peptides **15** and **16**, where substitution at the E-3 position (**15**) retained some moderate activity in comparison to the substitution of E-2 (**16**), again suggesting more promiscuity in the E-3 binding region of Hop_TPR2A_. Finally, it was interesting to note that the replacement of the V-4 residue of peptide **1** with proline (**17**) had a significantly detrimental effect on PPI disruption activity, in comparison to **1** while the proline analogue of **4** (**18**)^54^ was also inactive.

**Figure 4:**
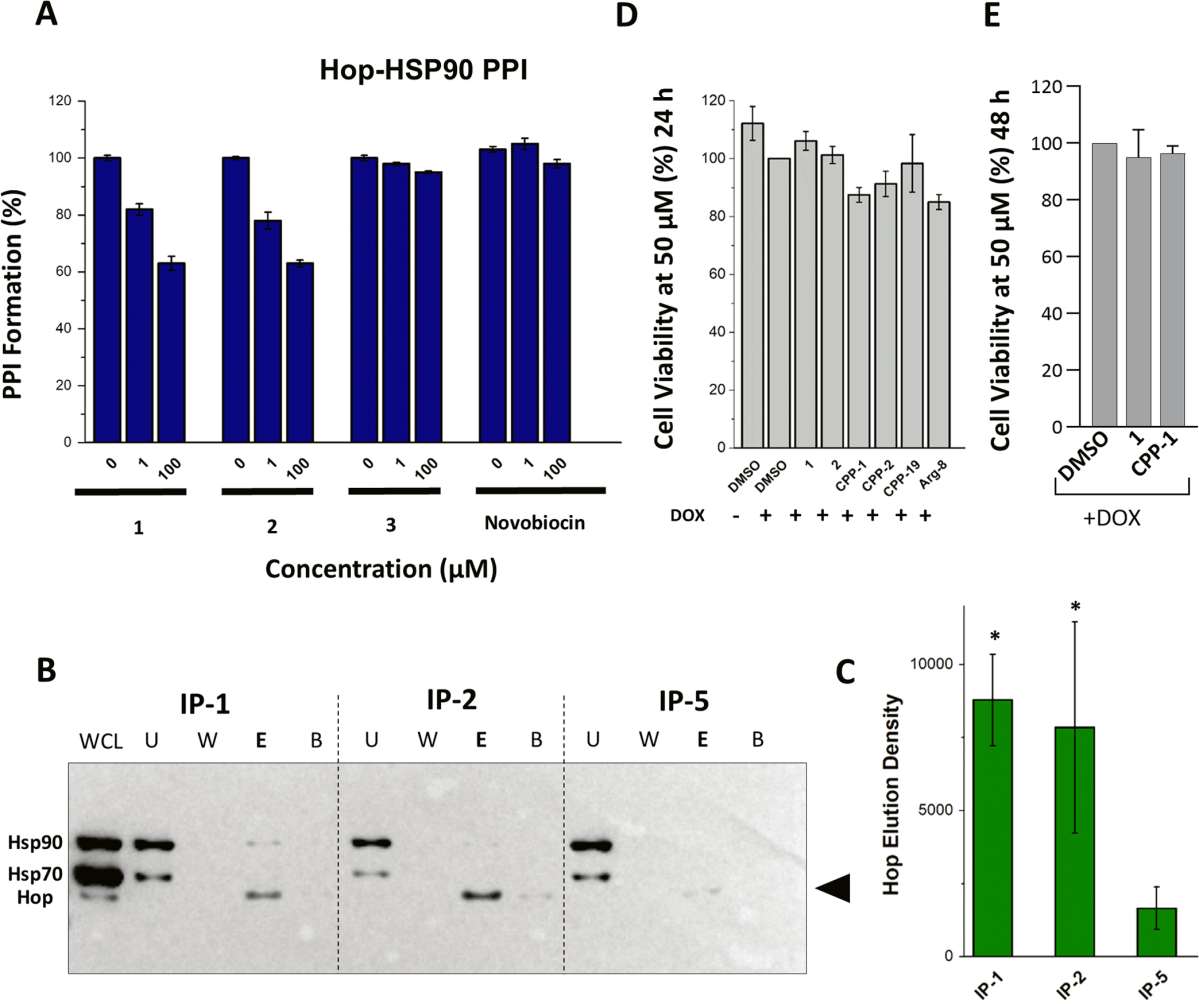
(A) Solid-phase Hop-HSP90 PPI inhibitory assay. Activity of **1** and **3** in the domain–domain interaction inhibition assay was retained against the full-length PPI. Statistical significance was determined by *t* test where *p < 0.01, **p < 0.001, ***p < 0.0005. (B) Western blot following immunoprecipitation of HeLa cell lysates with resin-supported peptides **IP-1** and **IP-2** indicate significant enrichment of Hop. In accordance with the weaker PPI inhibitory activity of peptide **5**, an immunoprecipitation experiment with **IP-5** resulted in trace quantities of Hop in the elution buffer. WCL: whole cell lysate(input), U: unbound fraction, W: wash, E: elution, and B: bead fraction. (C) Quantification of Hop elution through densitometry analysis points toward a relationship between PPI inhibition and Hop interaction in cell lysates. Cell viability of **1**, **2**, **CPP-1**, **CPP-2**, **CPP-19**, and Arg-8, against KSHV-infected TREx-BCBL-1-RTA and HEK293T cells, showed no significant cytotoxicity at 50 μM after incubation for (D) 24 and (E) 48 h respectively. Statistical significance was determined by unpaired, two-tailed *t* test where *p < 0.05.

Peptides **1** and **2** were identified as the most potent inhibitors of the Hop_TPR2A_–HSP90_CTD_ interaction in the solid-phase inhibitory assay, resulting in substantially reduced PPI formation at both 10 μM (25 and 18%) and 100 μM (16 and 11%), respectively (Figures 2 and 3). In a previous report by us,^45^ peptide **3** showed substantially weaker disruption of PPI formation at 10 μM (69%), while activity at 100 μM was more promising (26%). Accordingly, all three peptides were assessed for their ability to disrupt the full-length Hop-HSP90 PPI, again using an ELISA-based interaction assay (Figure 4A). Both **1** and **2** showed extremely encouraging dose-dependent activity in disrupting this large interfacial interaction. However, peptide **3** was found to be inactive in this assay presumably as a consequence of its weaker activity at the Hop_TPR2A_–HSP90_CTD_ interface.

### Immunoprecipitation and Hop Engagement in Cell Lysates

**Figure 5:**
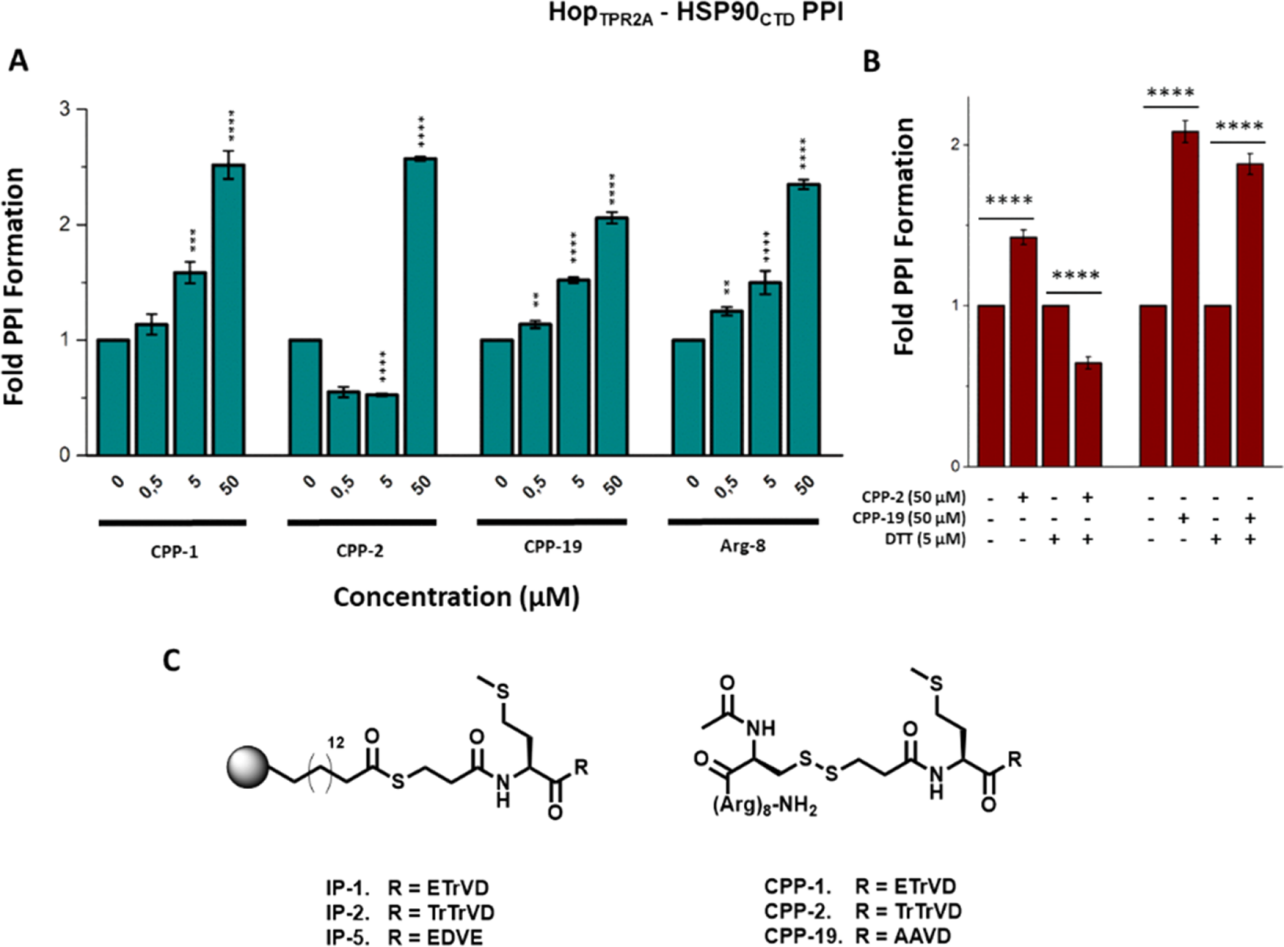
(A) Solid-phase Hop_TPR2A_–HSP90_CTD_ PPI inhibitory assay of **1**, **2**, **CPP-1**, **CPP-2**, **CPP-19**, and Arg-8. The presence of Arg-8 results in stabilization of the target PPI. (B) DTT-induced reductive release of the cargo peptide restores the PPI inhibitory activity of peptide **2**. (C) Modified peptides used in this study. Statistical significance was determined by unpaired, two-tailed *t* test where **p < 0.01, ***p < 0.005, and ****p < 0.001.

To augment the PPI inhibitory activity, we next sought to demonstrate the ability of inhibitory peptides to interact with Hop in a more biologically relevant context. The *N*-terminal acetyl of PPI disrupting peptides **1** and **2**, alongside the weakly active peptide **5** as a control, was immobilized on SulfoLink resin, to generate immobilized peptides **IP-1**, **IP-2**, and **IP-5** (Figure 5C). All three resin-supported peptides were incubated overnight with HeLa cell lysates, where, following standard procedures, eluted proteins were analyzed via Western blot (Figure 4B,C). Both HSP90 and HSP70 can be seen in high abundance in both the whole cell lysate as well as the input into the pulldown. Hop is present in lower abundance in the whole cell lysate and seemingly not detectable in the various inputs. However, incubation with **IP-1** and **IP-2** enriched the concentration of Hop in the elution buffer, indicating a strong interaction with Hop in the complex cell lysate matrix, while only trace amounts of Hop are seen in the control elution with **IP-5**, which correspond to its weaker PPI disrupting activity.

Equivalent assays were conducted in which HeLa Hop knockout cells were transfected with plasmids overexpressing either the Hop_TPR1_ or Hop_TPR2A_ domains. Both domains contain an EEVD binding region^50^ and could represent additional binding sites for peptides **1** and **2**. Here, **IP-2** only induced immunoprecipitation of Hop_TPR2A_ (Figure S1), indicating that the pull down of Hop as shown in Figure 4 occurs via selective interaction with Hop_TPR2A_ rather than Hop_TPR1_.

Interestingly, trace quantities of HSP90 were observed in the full-length Hop IP experiments involving **IP-1** and **IP-2**, suggesting that small quantities of HSP90 remained associated with Hop, following elution, likely via a weaker secondary interaction. This phenomenon was not observed with **IP-5**, presumably due to the comparatively lower concentration of Hop in the elution buffer. Given that HSP70 interacts with both Hop_TPR1_ and Hop_TPR2B_, the preference for which is influenced by the formation of the Hop_TPR2A_–HSP90_CTD_ PPI,^55^ it was noteworthy that trace HSP70 did not also appear in the Hop IP experiments.

### KSHV Lytic Replication Inhibition

**Figure 6:**
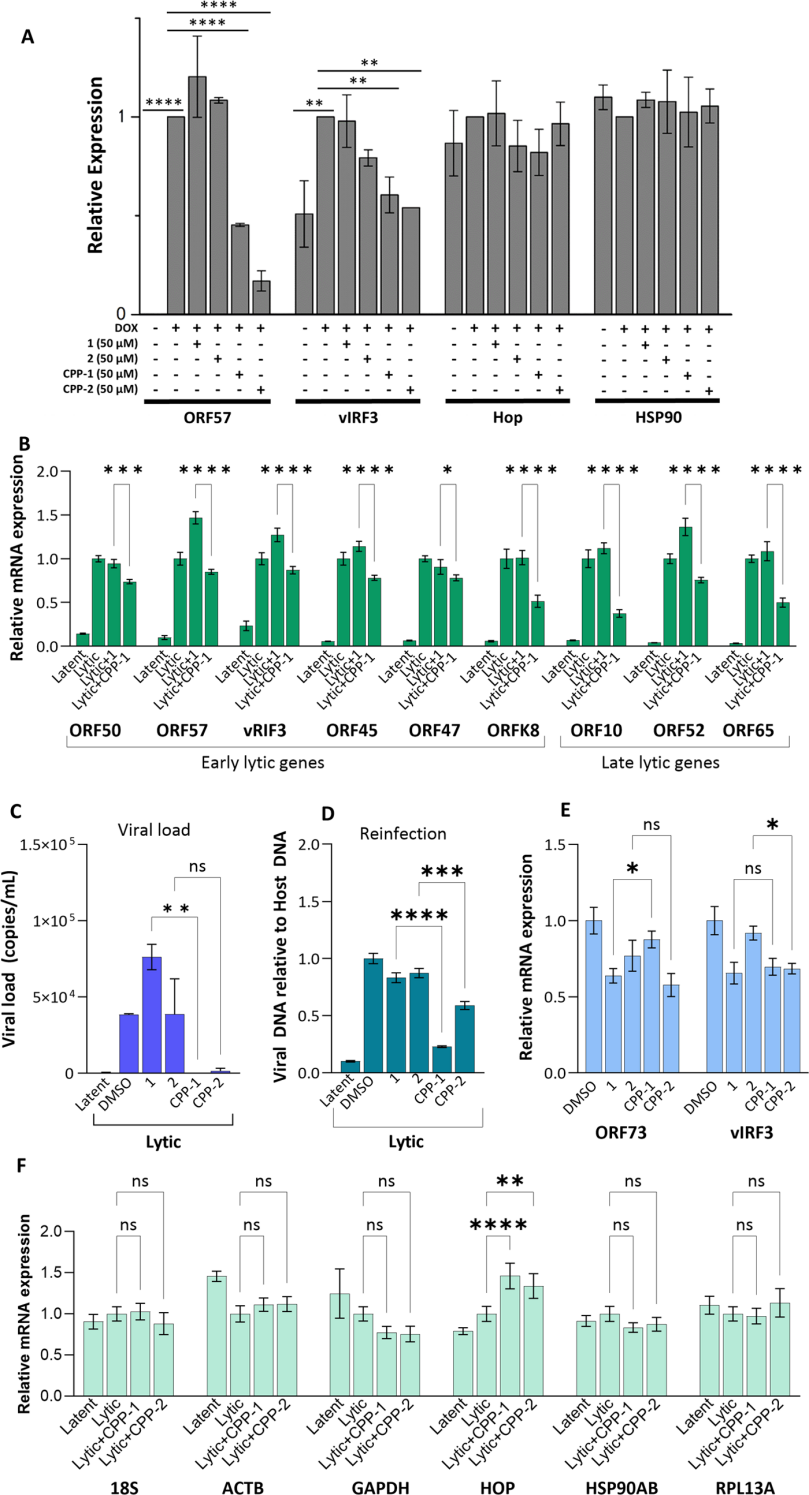
(A) Relative protein expression levels of early-stage lytic replication transcription factor, ORF57, and vIRF3 are inhibited by **CPP-1** and **CPP-2**. No effect was observed for the expression of Hop and HSP90. A representative Western blot is shown in Figure S2. (B) **CPP-1** significantly reduced the level of expression of multiple KSHV lytic genes. **CPP-1** and **CPP-2** significantly reduce (C) viral load in PEL cells and (D) production of infectious virions as measured by reinfection of naïve HEK293T cells. (E) **CPP-1** and **CPP-2** do not exert major changes to latent gene expression and (F) do not significantly alter host gene expression. Statistical significance was determined by ANOVA where *p < 0.05, **p < 0.01, ***p < 0.005, and ****p < 0.0001.

Hop is required for KSHV lytic replication.^38^ Having established full-length Hop-HSP90 PPI inhibitory activity in conjunction with Hop engagement in cell lysates, we focused on assessing the impact that PPI inhibitory peptides **1** and **2** would have on the activation of KSHV lytic replication. To address possible cell penetration limitations, we also generated Arg-8 CPP containing analogues of **1** (**CPP-1**,) and **2** (**CPP-2**), in addition to a MAAVD containing control (**CPP-19**, Figure 6C). Disulfide linkages to CPPs render them particularly useful as a pro-drug strategy, owing to the high concentration of glutathione in the cytosol, which facilitates the release of the cargo peptide from the CPP following cell penetration.^56^ To access our desired analogues, *N*-thiopropionic acid-modified peptides **1a**, **2a** and MAAVD analogue **19a** were tethered to the CPP via a labile disulfide linkage. In addition, Arg-8 was used as an additional control. To establish a noncytotoxic concentration window, peptides **1**, **2**, **CPP-1**, **CPP-2**, **CPP-19**, and Arg-8 were all assessed against the KSHV-infected TREx-BCBL-1-RTA cell line, where all the peptides displayed negligible cytotoxicity at 50 μM (Figure 4D,4E).

These peptides were then assessed for the ability to modulate the Hop_TPR2A_–HSP90_CTD_ interaction via the solid-phase binding assay. Peptides **CPP-1**, **CPP-19**, and Arg-8 stabilized the PPI in a dose-dependent fashion (Figure 5A). Interestingly, **CPP-2** still induced dose-dependent PPI disruption at lower concentrations (0.5 and 5 μM). However, at 50 μM, significant PPI stabilization was observed. As a proxy for intracellular glutathione, we repeated the assay with **CPP-2** and **CPP-19** in the presence of DTT, where the reductive release of the CPP restored the PPI disruption activity of **CPP-2**, while **CPP-19** remained inactive (Figure 5B).

KSHV ORF57 is an immediate gene that is expressed early during KSHV lytic replication. The protein product of this gene, mRNA transcript accumulation (Mta) protein, regulates RNA stability and the expression of other lytic genes, which promote viral transcription.^57,58^ In addition, KSHV viral interferon regulatory factor 3 (vIRF3), which plays a regulatory role in cell proliferation and apoptosis, as well as cellular IRF function,^17^ is one of the few genes expressed during latent replication, but whose expression increases during lytic replication. These proteins are, therefore, good markers for the activation of KSHV lytic replication. Accordingly, peptides **1**, **2**, **CPP-1**, and **CPP-2** were assessed for their ability to inhibit immediate early lytic replication by monitoring the protein levels of ORF57 and vIRF3, via Western blotting, as a proxy for KSHV lytic replication in reactivated TREx-BCBL-1-RTA cells.

Neither **1** nor **2** displayed any notable activity, while both **CPP-1** and **CPP-2** showed a statistically significant reduction in both the ORF57 and vIRF3 protein levels, with **CPP-2** performing slightly better than **CPP-1** (Figures 6A and S2).

In addition, none of the assayed peptides had any significant effect on the protein levels of either Hop or HSP90. **CPP-2** was further shown to inhibit ORF57 and vIRF3 expression in a dose-dependent fashion, while the control peptide **CPP-19** had no effect on either (Figure S3).

The subsequent qPCR analysis of KSHV mRNA expression showed that CPP**-1** significantly reduced the expression of several early and late lytic genes in TREx-BCBL-1-RTA PEL cells between 20 and 50% depending on the gene (Figure 6B). Treatment also significantly reduced viral load (Figure 6C) and infection of naïve HEK293T cells (Figure 6D). In contrast, the peptides did not significantly reduce the expression of latent genes ORF73 and vIRF3 (Figure 6E) or several host genes under lytic conditions (Figure 6F). The exception was the Hop transcript that was significantly upregulated with **CPP-1** or **CPP-2** treatment (Figure 6F). We speculate that this upregulation may reflect a cellular response to Hop inhibition. On this basis, our data suggest that the peptides do not act via a global reduction in transcription but rather through selective inhibition of specific KSHV genes. The antiviral effects were observed in the same time frame as the viability assessments (Figure 4B,4C) suggesting that reductions in lytic replications were not due to cellular cytotoxicity.

**Figure 7:**
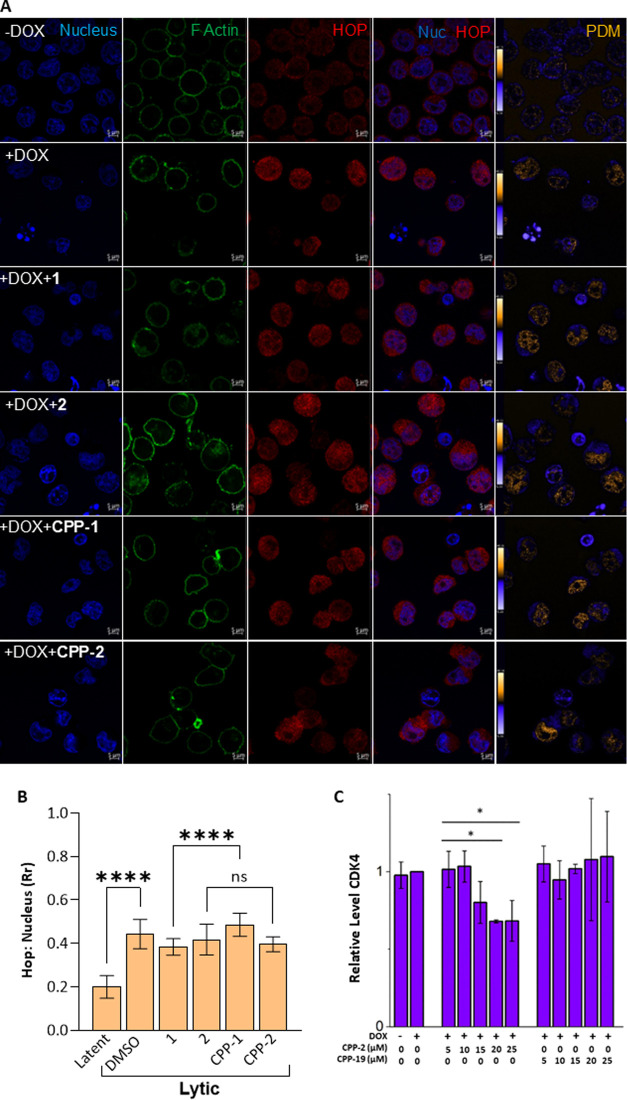
(A) Confocal microscopy of TREx-BCBL-1-RTA lines under latent and lytic conditions shows that **CPP-1** and **CPP-2** do not prevent Hop (red) recruitment to the nucleus (blue) during KSHV lytic replication. In panels labeled PDM, colocalization is indicated by positive correlations shown in yellow and confirmed by (B) the Pearson correlation coefficient (Rr) for the Hop and nuclear signals. (C) **CPP-2** reduces the abundance of HSP90 client CDK4, suggesting intracellular HSP90 inhibition. The data shown are averages ± SD (*n* ≥ 23) of the Pearson’s correlation coefficient between the Hop and nuclear signals in individual cells. Statistical significance was determined by ANOVA where *p < 0.05 and ****p < 0.0001.^60^

Finally, we attempted to gain a preliminary understanding of the mechanism of action of the peptides. Hop contains a nuclear localization signal (NLS)^59^ and is recruited to the nucleus upon KSHV lytic replication.^38^ We previously reported that peptide **1** when docked to TPR2A had an additional interaction between the tetrazole moiety and Lys237 of Hop.^45^ In addition to being in the interaction interface for Hsp90, Lys237 is part of the Hop NLS directing Hop to the nucleus.^59^ Therefore, we tested if the peptides could block Hop nuclear translocation culminating in the antiviral effect. As previously reported, we show that reactivation of lytic replication in TREx-BCBL-1-RTA cells promoted Hop nuclear localization (Figure 7A). Quantitative pixel-on-pixel colocalization analysis confirmed increased colocalization of Hop staining with the nuclear signal (Figure 7A, PDM), leading to an increased Pearson correlation coefficient between the two signals (Figure 7B, Rr).^60^ None of the peptides significantly blocked Hop nuclear translocation, suggesting that antiviral activity is not through the regulation of Hop subcellular distribution (Figure 7A,7B). However, the levels of HSP90 client CDK4 in reactivated TREx-BCBL-1-RTA cells were reduced in the presence of peptide **CPP-2**. This same effect was not seen with peptide **CPP-19** (Figures 7C and S4). Therefore, although we did not establish a causal link between PPI disruption and inhibition of KSHV lytic replication, **CPP-2** does appear to disrupt intracellular HSP90 function, which may, in part, underpin the biological activity against KSHV.

## Conclusions

The chaperome has a well-documented and essential role in maintaining cellular proteostasis in both healthy and diseased cells. This central function has seen numerous viral families evolve to parasitically exploit host chaperones in their life cycle, which has seen them emerge as promising host-based targets for antiviral drug discovery. Linked to this is the fundamental regulatory role that PPIs play in cellular biology, which not only offers the opportunity to develop our understanding of disease progression but also offers an underexplored chemical space for identifying new druggable targets.^61^ This latter point is particularly relevant for neglected diseases which have no well-defined druggable targets.^62^ To this end, recent advances in the Hsp90 drug discovery space, have seen a move toward designing molecules that modulate PPIs between Hsp90 and its cochaperones or client proteins as a means of achieving more selective Hsp90 regulation to target challenging diseases.^37^ This includes recent success in disrupting PPIs with pro-oncogenic clients such as B-Raf, CDK4, and c-SRC.^63^

In this study, which extended our previous discovery of potent Hop_TPR2A_ – HSP90_CTD_ inhibitory peptides **1** and **3**,^45^ we sought to further investigate disruption of the Hop-HSP90 PPI as a target for host-directed antiviral therapies. Here, we identified peptide **1**, and a double tetrazole-containing analogue **2** as *in vitro* inhibitors of the full-length Hop–HSP90 PPI, which were confirmed through immunoprecipitation analysis to interact with Hop in cell lysates. This interaction was further shown to be selective for Hop_TPR2A_ over Hop_TPR1_. Arg-8-modified analogue **CPP-2** was found to inhibit intracellular HSP90 function, and **CPP-1** and **CPP-2** functioned as noncytotoxic inhibitors of KSHV lytic replication by blocking gene expression, coinciding with reduced viral load and reduced infectious virions. Furthermore, our data indicated that this activity did not occur via generalized inhibition of transcription but rather because of selective inhibition of specific KSHV lytic genes. HSP90 is a known chaperone for numerous viral transcription factors,^8,24^ and by extrapolation, it is possible that the biological factors responsible for lytic gene expression are HSP90 clients. It is critical to note, however, that while we have individually demonstrated Hop–HSP90 PPI inhibition, Hop engagement in cell lysate, and HSP90 inhibition, we have yet to explicitly link these three phenomena to each other. An alternative explanation is that Hop itself plays a regulatory role in the transcription of viral genes, either directly via the Hop_TPR2A_ domain or indirectly via interaction with HSP90. If this were the case, then the observed activity would not entirely be a result of HSP90 inhibition.

Therefore, while it is premature to pronounce a pharmacological validation of the PPI as a chaperone inhibiting anti-KSHV target, these probe peptides represent a tantalizing correlation between KSHV lytic replication and Hop-HSP90 assembly and, as such, are an important step forward in understanding host HSP90–viral interactions. Together, this provides further evidence for Hop-HSP90 PPI as a much-needed target for KSHV drug discovery and possibly other closely related herpesviruses.

## Methods

All of the reagents and solvents were obtained from commercial suppliers and used without further purification. CTC-resin and the protected amino acids were purchased from Iris Biotech. DIC and OxymaPure were a gift of Luxembourg Biotech and *N*,*N*-diisopropylethylamine (DIEA) from Sigma-Aldrich. Organic solvents [DMF and acetonitrile (CH3CN)] were purchased from Merck. Reversed-phase HPLC (RP-HPLC) quality CH3CN and Milli-Q water were used for the RP-HPLC analyses. SulfoLink Coupling Gel, Pierce, Rockford, IL. Analytical HPLC was performed on an Agilent 1100 system using a Phenomenex C18 column (3 μm, 4.6 mm × 50 mm), and Chemstation software was used for data processing over a 5–95% gradient (otherwise a different gradient is stated) of ACN (0.1% TFA)/H_2_O (0.1% TFA) over 15 min at a flow rate of 1.0 mL/min and detection at 220 nm at 30 °C. All mass spectrometry data were obtained from a Thermo Fisher Scientific UltiMate 3000 UHPLC-ISQTM EC single quadrupole mass spectrometer in positive ion mode over a 5–95% gradient (otherwise a different gradient is stated) of ACN (0.1% HCOOH)/H_2_O (0.1% HCOOH) for 15 min.

### Solid-Phase Peptide Synthesis

Manual syntheses were carried out in a polypropylene syringe fitted with a porous polyethylene disc and attached to a vacuum trap for easy filtration. Syntheses were done at 0.1 mmol scale on 2-chlorotrityl chloride (2-CTC) resin with a nominal loading of 1.2 mmol/g, which was activated with 50% thionyl chloride in DCM for 2h. First amino acid coupling was done with Fmoc-Leu-OH (3 equiv) and DIEA (*N*,*N-*diisopropylethylamine) (10 equiv) in DCM for 2 h; then, for capping the unreacted Cl groups, MeOH (50 μL) was added and allowed to react for 30 min at room temperature. Fmoc was removed by treatment of the resin twice with a solution of 20% piperidine in DMF for 1 + 7 min. The remaining couplings were done with a 3-fold excess of Fmoc-amino acid, DIC, and OxymaPure in a 1:1:1 ratio for 45 min. After the peptide chain elongation, peptides **2**, **5–18** were acetylated at the N-terminal by treatment with 10 equiv of acetic anhydride/diisopropylethylamine (1:2) in DMF for 30 min. Peptides **IP-1**, **IP-2**, **IP-5 CPP-1**, **CPP-2**, and **CPP-19** were all generated via a mercaptopropyl-derivative (Mpa). Mpas were made by reaction of the appropriate nonacetylated peptide with 3,3′-dithiodipropionic acid (DTDP; 3 equiv) using DIC (3 equiv) and OxymaPure (3 equiv) in DMF for 30 min; then, before cleavage, each intermediate was reduced to the free thiol by three treatments of 10 equiv of DTT each in a DMF/DIEA/H2O sol (92:4:4) mixture for 10 min each. Cleavage was performed after drying the peptidyl resins by treating with TFA/TIS/H_2_O (95:2.5:2.5) for 1h at rt. The cleavage mixture was then precipitated with chilled Et_2_O, centrifuged, and the pellet was redissolved for analysis by HPLC and LCMS. All peptides were purified by semipreparative RP-HPLC to purity >95%. Please see Figures S5–S9 in the Supporting Information file.

### Immobilization of Thiol-Derivatized Peptides to Agarose (SulfoLink)

SulfoLink Gel Columns were prepared on plastic columns by loading 2 mL of gel slurry to obtain a 1 mL gel bed. The peptide immobilization was done following the manufacturers instructions. Briefly, a 2 mL solution of thiol-peptide (3 mg/mL) in 50 mM Tris/5 mM EDTA buffer at pH 8.50 was made to react with iodoacetyl-modified agarose for 45 min at room temperature (first 15 min with stirring). The coupling was confirmed by HPLC analysis of the coupling solution before and after the reaction (Figure S5). After the columns were washed with 1 M NaCl, any remaining iodoacetyl groups in the agarose were blocked by cysteine solution (50 mM, under the same conditions described above). Please see **Figure S10** in the Supporting Information file.

### Conjugation of Thiol-Derivatized Peptides to the Cell-Penetrating Peptide (CPP) Arg_8_

Arg_8_ was synthesized manually by SPPS as explained above except for the incorporation of a Rink amide resin was used in this instance.^64^ The sequence was modified at the N-terminus by adding a Cys(SIT) residue to direct the disulfide conjugation and acetylation of the amino group. The conjugation of Ac-Cys(SIT)-R_8_-NH_2_ and Mpa-derivatized peptides was done in solution with a molar ratio of 1:1. Ac-Cys(SIT)-R_8_-NH_2_ was dissolved in aqueous NH_4_HCO_3_ (10 mM) to a concentration of 1 mM; to this solution, the Mpa-peptide dissolved in H_2_O (5 mM) was added and vigorously stirred at RT. The reaction was completed in 30 min (HPLC monitored), then the solution was quenched by the addition of AcOH, and the product was lyophilized. Please see Figures S11–S13 in the Supporting Information file.

### Cell Culture

TREx-BCBL-1-RTA cells (a generous gift from Prof. Jae Jung, University of Southern California) are a KSHV-infected BCBL-1-based primary effusion lymphoma B-cell line, which expresses Myc-tagged RTA upon induction with doxycycline. TREx-BCBL-1-RTA cells were cultured in RPMI 1640 medium (Thermo Fischer) supplemented with 10% (v/v) fetal bovine serum (FBS) (Gibco, UK), 1% (v/v) GlutaMAX-I (GibcoTM, Life Technologies), and 1% (v/v) penicillin/streptomycin/amphotericin (PSA) (Lonza Group Ltd., Basel, Switzerland). The cell line was kept under hygromycin B (Glentham Life Sciences, UK) selection (100 μg/mL) and was cultured at 37 °C in a humidified incubator with 5% CO_2_. KSHV lytic replication was induced in TREx-BCBL-1-RTA cells by the addition of 2 μg/mL DOX. The HeLa cervical carcinoma cell line (CHLA-FL) (Cellonex) and HeLa HOP knockout cell line (developed using CRISPR/Cas9 technology as part of another study) were cultured in DMEM supplemented with 10% (v/v) FBS, Glutamine (100 U/mL), and PSA (100 U/mL). HEK293T cells (ATCCCRL-3216) were purchased from ATCC. HEK293T is a human embryonic kidney-derived cell line immortalized by transformation with SV40 T-antigen.^65^ HEK293T cells were cultured in Dulbecco’s Modified Eagle Medium (DMEM) (LTC Tech, Johannesburg, South Africa) supplemented with 10% (v/v) FBS, 1% (v/v) sodium pyruvate (Sigma-Aldrich, Darmstadt, Germany), 1% (v/v) nonessential amino acids (Lonza, BE13–114E), 1% (v/v) PSA, 1% (w/v) G418 (Glentham Life Sciences, GA2946), and 1% (v/v) GlutaMAX-I at 37 °C in a humidified incubator with 9% CO_2_.

### Hop-Hsp90 Protein–Protein Interaction Assay

ELISA-based Hop-Hsp90 PPI solid-phase binding assays were performed in microtiter 96-well flat bottom high-binding plates (Greiner Bio-One, Germany) according to our previously published method with modifications.^48^ Briefly, 50 μL of 2.5 μM purified Hop or Hop_TPR2A_ diluted in binding buffer A (20 mM Tris-HCl (pH 7.4), 150 mM NaCl, and 0.05% (v/v) Tween-20) was incubated at 4 °C for 16 h in wells of a high-binding plate. Any unbound protein was discarded and 300 μL of 3% (w/v) BSA in buffer A was used to block nonspecific binding sites in the wells by incubating at 25 °C for 2 h. The BSA was discarded, and 50 μL of the peptides (diluted in buffer A at desired final concentration(s)) or DMSO (diluted in buffer A to serve as the untreated control for 100% PPI) was incubated at 4 °C for 16 h in the wells. A total of 50 μL of 0.9 μM purified Hsp90 or GST-Hsp90_CTD_ (diluted in binding buffer A) was added into each well and incubated at 4 °C for 16 h. The wells were washed three times with 200 μL of 1% (w/v) BSA in buffer A. A total of 50 μL of mouse anti-Hsp90α/β (F-8) monoclonal antibody (Santa Cruz, sc-13119) or mouse anti-GST monoclonal primary antibody (B-14) (Santa Cruz, sc-138) diluted in buffer A at 1:5000 was added to each well, and the mixture was incubated at 4 °C for 16 h. The wells were washed thrice with 200 μL of 1% (w/v) BSA in buffer A. A total of 50 μL of horseradish peroxidase (HRP)-conjugated donkey antimouse IgG secondary antibody (Thermo Fisher Scientific, A16011) diluted in buffer A at 1:5000 was added to each well and incubated at 25 °C for 1 h. The wells were washed thrice with 200 μL of 1% (w/v) BSA in buffer A. A volume of 100 μL of HRP buffer (25.7 mM citric acid, 48.6 mM Na_2_HPO_4_, 1 mg/mL TMB (in DMSO), and 30% (v/v) H_2_O_2_, pH 5) was pipetted into each well and the plate was incubated at 25 °C for 15 min in the dark. To stop the reaction, 50 μL of 1 M Sulfuric acid was added to each well. A BioTek Epoch 2 Microplate Spectrophotometer (BioTek, Vermont) using Gen5 software was used to measure the intensity of the yellow color at 450 nm. The absorbance value is proportional to the level of Hop – Hsp90 or Hop_TPR2A_ – GST-Hsp90_CTD_ PPI.

### Cell Viability Assessment

Resazurin cell viability assay was used to determine the cytotoxicity and cell viability. Briefly, 2 × 10^6^ TREx-BCBL-1-RTA cells, at a seeding density of 4 × 10^5^ cells/mL, were supplemented with either 0.25% DMSO (as unreactivated DMSO control) or 0.25% DMSO and 2 μg/mL DOX (as the reactivated DMSO control) or 2 μg/mL DOX and 50 μM of each peptide for 22 h. Each sample was resuspended properly, and 100 μL of each cell suspension was transferred into triplicate wells of a flat 96-well tissue culture plate, while the remaining cell suspension was subsequently used for Western blot analysis. For HEK293T cells, the cells were seeded at a density of 1.5 × 10^5^ cells/mL in the medium in wells of a flat 96-well tissue culture plate a day before treatment (to allow the cells enough time to attach to the plate). The cells were treated with either 0.25% DMSO (as an untreated control) or 50 μM of each peptide for 46 h. Resazurin dye (7-hydroxy-3H-phenoxazin-3-one 10-oxide) was added to the TREx-BCBL-1-RTA or HEK293T cells and incubated at 37 °C for 2 h. Cell viability was determined by measuring the fluorescence intensity at excitation and emission wavelengths of 560 and 590 nm, respectively. The relative cell viability for the treated cells was calculated as a percentage of fluorescence reading for treated cells compared to fluorescence reading for the reactivated DMSO control.

### Western Blotting

The remaining TREx-BCBL-1-RTA cells (after cell viability assay) were harvested and washed twice with ice-cold PBS (pH 7.4). The cells were lysed on ice for 20 min using radioimmunoprecipitation assay (RIPA) lysis buffer (Sigma-Aldrich, Germany) supplemented with 1× cOmplete EDTA-free protease inhibitor cocktail (Sigma-Aldrich, Germany) to extract protein samples from the cells. Bicinchoninic acid (BCA) protein quantification assay was used to determine the concentration of protein in the lysates. An equal amount of protein samples was denatured in Laemmli SDS loading buffer at 95 °C for 5 min. An equal volume of the denatured protein samples was resolved by SDS-PAGE and transferred to nitrocellulose membranes using the complete Bio-Rad Trans-Blot Turbo Transfer System (Bio-Rad, California). The membranes were blocked in 5% (w/v) Blotto (Santa Cruz Biotech, San Diego, CA) dissolved in Tris-buffered saline containing 0.1% Tween-20 (TBST). The membranes were incubated in an appropriate primary antibody. The primary antibodies used in this study were mouse anti-ORF57 (207.6) monoclonal antibody (Santa Cruz Biotechnology, sc-135746), mouse anti-LANA2/vIRF3 (CM-A807) monoclonal antibody (Novus Biologicals, NB200-167SS), rabbit anti-Hop monoclonal antibody (Abcam, ab126724), mouse anti-Hsp90α/β (F-8) monoclonal antibody (Santa Cruz, sc-13119), anti-CDK4 antibody [EPR4513-32-7] (Abcam, Ab108357), rabbit anti-β-actin [EPR2124] monoclonal antibody (Abcam, Ab213262), and HRP-conjugated anti-GAPDH antibody (EPR6256) (Abcam, ab185059). The membranes were washed three times using TBST and incubated in an appropriate secondary antibody. The secondary antibodies used in this study were HRP-conjugated donkey antirabbit IgG (Thermo Fisher Scientific, A16023) and HRP-conjugated donkey antimouse IgG (Thermo Fisher Scientific, A16011). Visualization of blots and image capture were performed using the Clarity Western ECL substrate kit (Bio-Rad, California) and Bio-Rad ChemiDoc MP System (Bio-Rad, California), respectively.

### Pulldown Using Peptide-Resins

Cell lysates from HeLa wild type or HeLa HOP knockout cells transfected with expression plasmids for HOP HA-TPR1, HA-TPR2A, or HA-TPR2B domains were prepared in RIPA buffer. A total of 500 μg of lysate in 500 μL was incubated with 25 μL of peptide-conjugated resin at 4 °C overnight with rotation. The following day, the beads were washed 7 times with PBS and bound proteins eluted with hot SDS loading buffer and incubated at 77 °C for 5 min before analysis by SDS-PAGE and Western blot analysis.

### Viral DNA Quantitation

TREx-BCBL-1-RTA cells, at a seeding density of 4 × 10^5^ cells/mL, were supplemented with either 0.25% DMSO (as an unreactivated DMSO control) or 0.25% DMSO and 2 μg/mL DOX (as the reactivated DMSO control) or 2 μg/mL DOX and 50 μM of each peptide for 72 h. Zymo Research *Quick*-DNA Miniprep kit (Zymo Research, cat. No.: D3025) was used to extract genomic DNA from the cells according to the manufacturer’s instructions. The genomic DNA was quantified, and 10 ng was used for qPCR analysis with primers specific to the KSHV ORF57 gene (to assess the level of viral DNA) and the human GAPDH gene (as the normalizer). Comparisons were made between the viral DNA levels in the peptides-treated reactivated TREx-BCBL-1-RTA cells and the viral DNA levels in the reactivated DMSO control TREx-BCBL-1-RTA cells.^66^

### Viral and Host mRNA Quantitation

For viral lytic and host mRNA quantitation in the PEL cell line, TREx-BCBL-1-RTA cells, at a seeding density of 4 × 10^5^ cells/mL, were supplemented with either 0.25% (v/v) DMSO (as unreactivated DMSO control) or 0.25% (v/v) DMSO and 2 μg/mL DOX (as the reactivated DMSO control) or 2 μg/mL DOX and 50 μM of each peptide for 24–48 h. For viral latent mRNA quantitation in the PEL cell line, TREx-BCBL-1-RTA cells, at a seeding density of 4 × 10^5^ cells/mL, were supplemented with either 0.25% (v/v) DMSO (as untreated control) or 50 μM of each peptide for 24 h. Following the manufacturer’s instructions, the Zymo Research Direct-zol RNA Miniprep Plus kit (Zymo Research, cat. No.: R2073) was used to extract total RNA, and the Zymo Research RNA Clean and Concentrator-5 kit (Zymo Research, Catalogue number: R1014) was used to remove viral and/or genomic contamination from the RNA samples through DNase I treatment. LunaScript RT SuperMix kit (New England Biolabs, cat. No.: E3010L) was used to reverse transcribe the cleaned-up RNA to cDNA according to the manufacturer’s instructions. The cDNA was subjected to qPCR analysis, and the expression levels of viral mRNA transcripts relative to human RPL13A mRNA transcript (used as a normalizer) were determined. The forward and reverse sequences of the oligonucleotide primer sets used in this study for qPCR are ORF57 (5′-CACTTCTGGAATACTACAGGCCAGG-3′; 5′-GTTAAATTTGGCCGACCCCATTGC-3′), ORF47 (5′-CGCGACCACTGCAGATAGCTCTATTC-3′; 5′-TTCCCTTTTGACCTGCGTGCG-3′), ORF65 (5′-CCTTAGGGATCAGAAACCGCGC-3′; 5′-CAGAGGACAGGGTGGTTATACTGC-3′), ORF45 (5′-CCGCCCACTCGATTTCATCAGG-3′; 5′-TCCAGCCACGGCCAGTTATATGC-3′), ORFK8 (5′- GTCATCGAAAGCATACACAAGACAGC-3′; 5′- TGCTGGCACATTCGCATCAGC-3′), ORF10 (5′- ATCTCTAATCGAGTACCCCT-3′; 5′- TAGAATGCAGTGTAGGTCTC-3′), ORF52 (5′- ACCTTGCTGAATATTGTCCT-3′; 5′- CCTTACGATGGAAGACCTAA-3′), ORF50 (5′-AGACCCGGCGTTTATTAGTACGT-3′; 5′-CAGTAATCACGGCCCCTTGA-3′), ORF73 (5′- CCAGACCAGTCGCCCATAACTTATTG-3′; 5′-GGAAGATTGTAGGTTTCCTGCCAGG-3′), vIRF3 (5′-AGCCGTACACTGTGTTGATAC-3′; 5′-CACGATTCATAGTGAGAAACA-3′), 18s rRNA (5′-GGATGTAAAGGATGGAAAATACA-3′; 5′-TCCAGGTCTTCACGGAGCTTGTT-3′), HOP1B (5′- GTCCCGGTAGCTTCTAGTAGGTTCC-3′; 5′-GGCCTTGTTGCCTTTCTCCTTC-3′), HSP90AB1 (5- CATCATCAATACCTTCTATTCCAACA-3′; 5-TTCAGCTCTTTACCACTGTCC-3′), ACTB (β-actin) [IDT: Hs.PT.39a.22214847], RPL13A (5′-GGCTTTCCTCCGCAAGCGGAT-3′; 5′-GCAGCATACCTCGCACGGTCC-3′) and GAPDH [IDT: Hs.PT.39a.22214836 (5′-ACATCGCTCAGACACCATG-3′; 5′-TGTAGTTGAGGTCAATGAAGGG-3′)]. The primer sets were purchased from Integrated DNA Technologies.

The CFX Connect Real-Time PCR Detection System (Bio-Rad, SA) was used to run the qPCR. The number of controls and replicates used for qPCR in this study was in conformity with the minimum information for publication of quantitative real-time PCR experiments (MIQE) guidelines.^67^ The Luna Universal qPCR Master Mix Kit (New England Biolabs, cat. No.: M3003) was used to set up the qPCR reactions according to the manufacturer’s instructions. The thermocycling protocol used was the initial denaturation step (a cycle at 95 °C for 60 s), denaturation step (40 cycles at 95 °C for 15 s/cycle), and extension step (40 cycles at 60 °C for 30 s). Melt curve analysis was performed for each run at a 0.2 °C increment from 60 to 95 °C to confirm that a single specific product was amplified. The CFX Maestro Software (Bio-Rad, SA) was used to analyze the qPCR data. The genes of interest were normalized to GAPDH and their relative expression levels were determined using the comparative C_*T*_ (ΔΔC_*T*_) method.^68^ Comparisons were made between the viral transcript levels in the peptide-treated reactivated TREx-BCBL-1-RTA cells and the viral transcript levels in the reactivated DMSO control TREx-BCBL-1-RTA cells.

### Viral Reinfection Assay and Determination of Viral DNA Copy Number

The viral reinfection assay was performed to determine the KSHV infectivity of naïve HEK293T cells by reactivated TREx-BCBL-1-RTA cells. The viral reinfection assay was performed as previously described with slight modifications.^66^ Briefly, TREx-BCBL-1-RTA cells, at a seeding density of 1 × 10^6^ cells/mL, were supplemented with either 0.25% (v/v) DMSO (as the unreactivated DMSO control), 0.25% (v/v) DMSO and 2 μg/mL DOX (as the reactivated DMSO control), or 2 μg/mL DOX and 50 μM of each peptide for 72 h. The cell suspensions were centrifuged at 500*g* for 5 min at 4 °C. The cell pellets were washed with ice-cold PBS and used for indirect immunofluorescence assay (IFA). The supernatants were filtered using 0.45 μm filters. The filtered supernatants were split and used to infect naïve HEK293T cells and to determine the viral DNA copies in each viral supernatant. A 1 mL volume of each filtered viral supernatant was used to infect HEK293T cells (that had been seeded in a 12-well plate the day before) for 24 h. Genomic DNA was extracted from the infected HEK293T cells, and qPCR analysis was used to determine the expression level of KSHV ORF57 gene in the HEK293T cells as a measure of KSHV infection. To determine the viral DNA copies/mL of each viral supernatant, a 2 μL volume of each filtered viral supernatant was used for qPCR analysis with primers specific to the KSHV ORF57 gene. The amount of KSHV DNA in ng was determined from a standard curve and converted to viral DNA copy number using the equation (ng RNA × 6.022 × 10^23^)/(length × 1 × 10^9^ × 660).

### Indirect Immunofluorescence Assay (IFA) and Confocal Microscopy

The nuclear localization of Hop in reactivated TREx-BCBL-1-RTA cells treated with 50 μM of each peptide was determined by IFA and confocal microscopy. Briefly, 5 × 10^5^ cells in 1 mL of PBS were incubated at 37 °C for 30 min on poly-l-lysine-coated coverslips in 24-well plates. The PBS was aspirated, and the attached cells were fixed with 4% (w/v) paraformaldehyde at room temperature for 10 min. The cells were permeabilized with 0.1% Triton X-100 at room temperature for 15 min. The cells were washed thrice with PBS and incubated in blocking solution (1% w/v BSA in PBS) at room temperature for 45 min. The cells were incubated in primary antibodies anti-HOP/STI1 (sc390203, 1:100 dilution) and Actin Green 488 (R37110, 1:100 dilution) at 4 °C for 16 h. The cells were washed thrice with PBS with 0.1% (v/v) Tween-20 (PBST) and incubated in fluorescently conjugated donkey antimouse IgG H&L (Alexa Fluor 555) secondary antibody (Ab150106, 1:1000 dilution) at room temperature for 1 h in the dark. The cells were washed twice in PBST and once in 1 μg/mL Hoechst 33342 (H1399) for nuclear staining. The coverslips were mounted with a DAKO fluorescent mounting medium. Images were captured using a Zeiss LSM980 confocal microscope with Airy scan 2. Quantitative colocalization analysis was performed using the Intensity Correlation Analysis plugin in ImageJ.^60^

## Supporting Information Available

Western blot and peptide characterization data (PDF)
