# Supplementary material for: Modulators of the Hop-HSP90 Protein–Protein Interaction Disrupt KSHV Lytic Replication
Source: ACS Infect Dis. 2024 Oct 30;10(11):3853–67. doi: 10.1021/acsinfecdis.4c00429 (PMC11555673; doi:10.1021/acsinfecdis.4c00429)
Supplement: Supplementary file 1 — id4c00429_si_001.pdf [file id4c00429_si_001.pdf]

Supporting Information

Modulators of the Hop-HSP90 Protein-Protein Interaction Disrupt KSHV Lytic Replication

Michael O. Okpara[1], Michaelone C. Vaaltyn[1], Jessica Watson[1], Mahama Alhassan[2], Fernando Albericio[2], Beatriz G. de la Torre[3], David J. Clarke[4], Clinton G. L. Veale[5*], Adrienne L. Edkins[1*]

[1]Biomedical Biotechnology Research Unit (BioBRU), Department of Biochemistry and Microbiology, Rhodes University, Makhanda, 6139, South Africa.

[2]School of Chemistry and Physics, University of Kwa-Zulu Natal, Westville, 4001, South Africa

[3]School of Laboratory Medicine and Medical Sciences, University of Kwa-Zulu Natal, 4041, South Africa

[4]EaStCHEM, School of Chemistry, University of Edinburgh, Joseph Black Building, David Brewster Road, Edinburgh, EH93FJ

[5]Department of Chemistry, University of Cape Town, Rondebosch, Cape Town, 7701, South Africa.

*Correspondence: clinton.veale@uct.ac.za, a.edkins@ru.ac.za

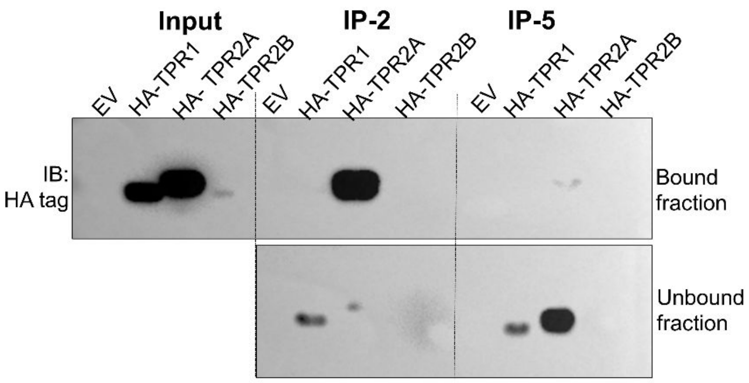

Figure S1. Selectivity of peptide 2 for TPR2A domain determined by pulldown assay using resin-conjugated peptides. HOP knockout cell lysates expressing HA-TPR1, HA-TPR2A or HA-TPR2B were incubated with resin-immobilised peptides 2 and 5. Proteins associating with the peptide-bound resin (bound fraction) or remaining in solution (unbound fraction) were detected after elution by SDS-PAGE and western blot analysis using an anti-HA antibody.

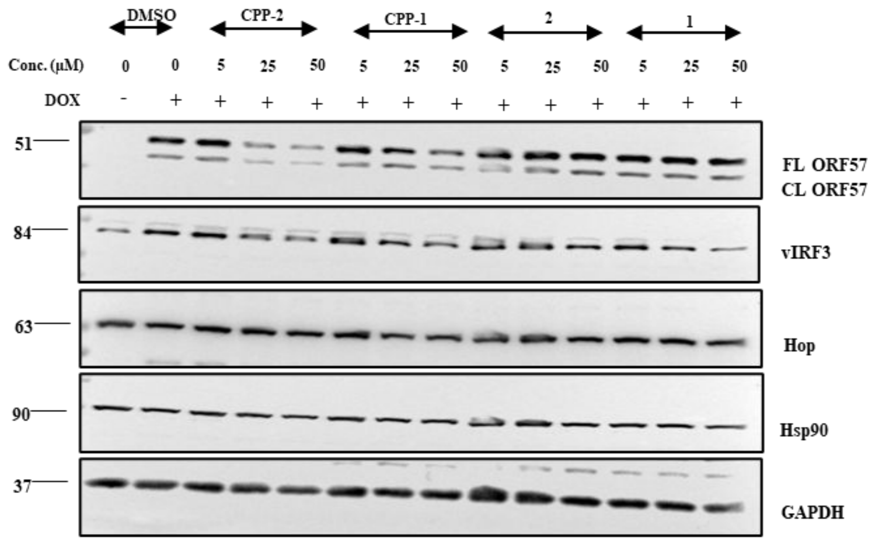

Figure S2. Initial Western blot analysis for effect of **CPP-1, CPP-2, 1 and 2** on KSHV ORF57, vIRF3, Hop, & Hsp90 protein expression in reactivated TREx-BCBL-1-RTA cells.

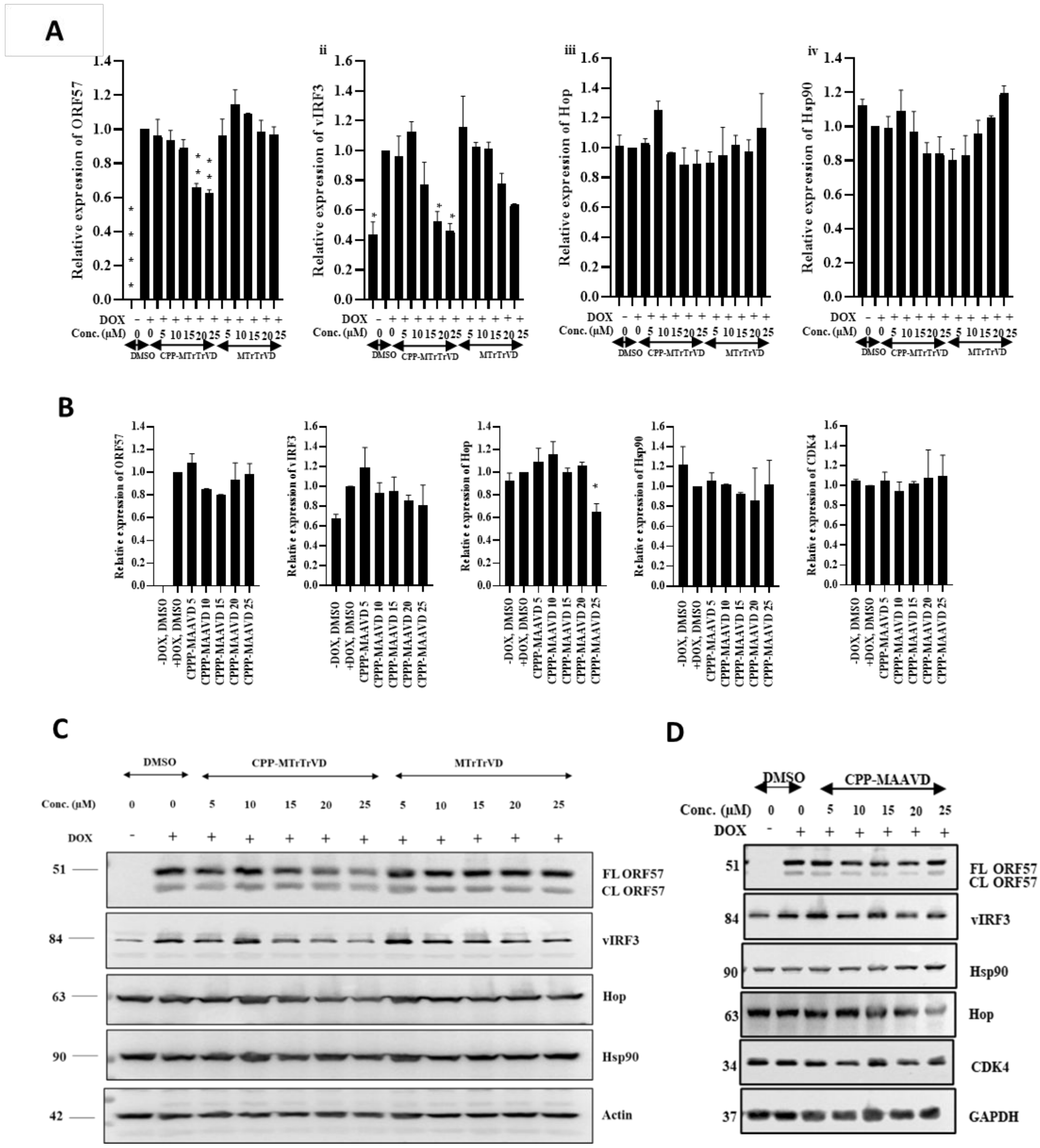

Figure S3. Densitometry (**A and B**) and Western blot analysis of expanded dose-dependent analysis (**C and D**) for CPP-MTrTrVD (**CPP-2**), MTrTrVD (**2**) and CPP-MAAVD (**CPP-19**) on KSHV ORF57, Virf3, Hop, & Hsp90 protein expression in reactivated TREx-BCBL-1-RTA cells.

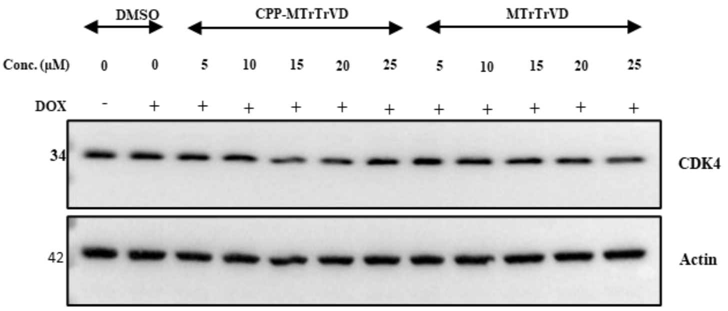

Figure S4. Western blot analysis of CPP-MTrTrVD (**CPP-2**) and MTrTrVD (**2**) on CDK4 protein levels in reactivated TREx-BCBL-1-RTA cells.

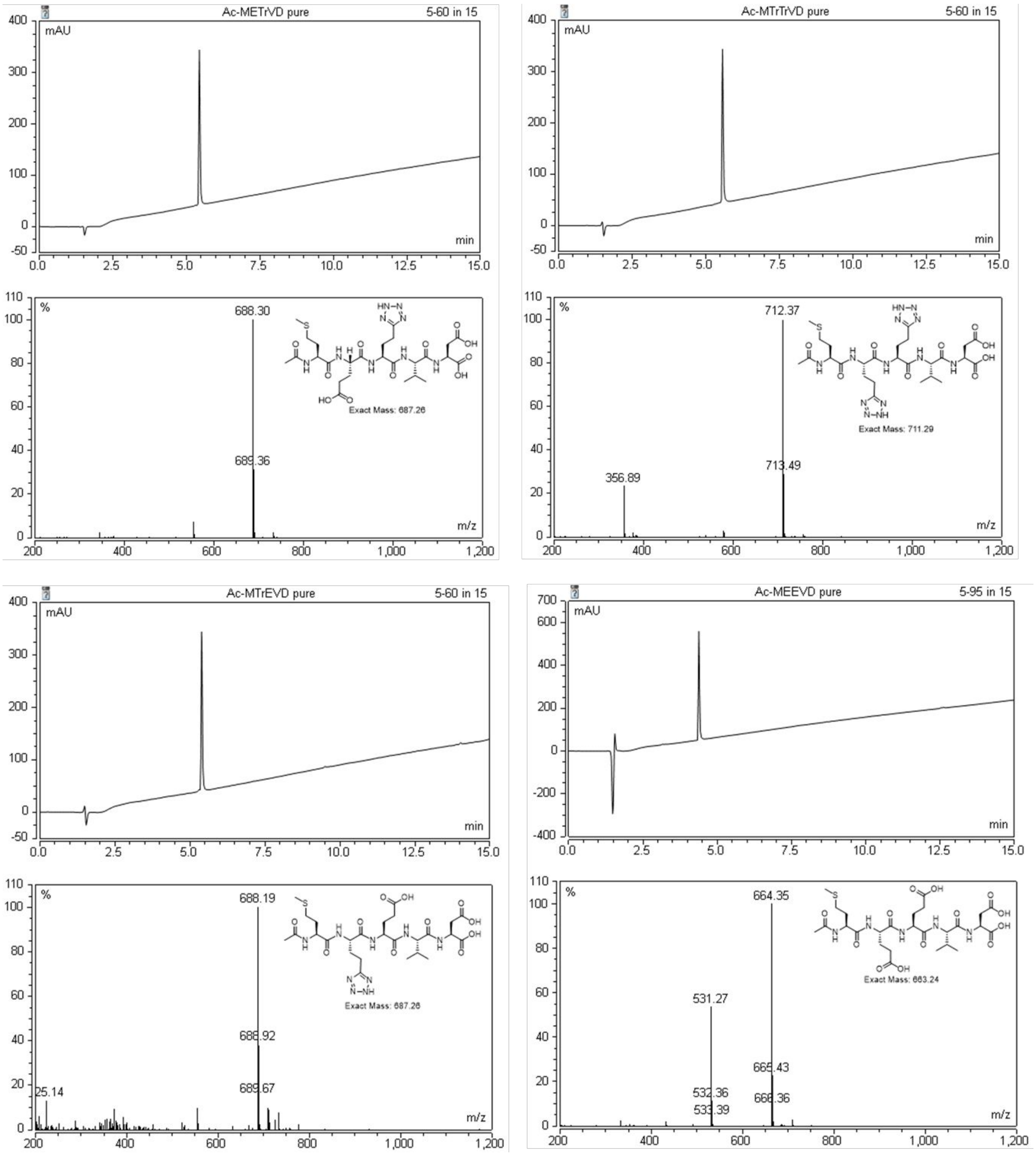

Figure S5. LC-MS analysis of peptides **1 – 4**. Upper panel: UV detector at 220nm. Lower panel: experimental mass in postive ion mode $[M+1H]^{1+}$

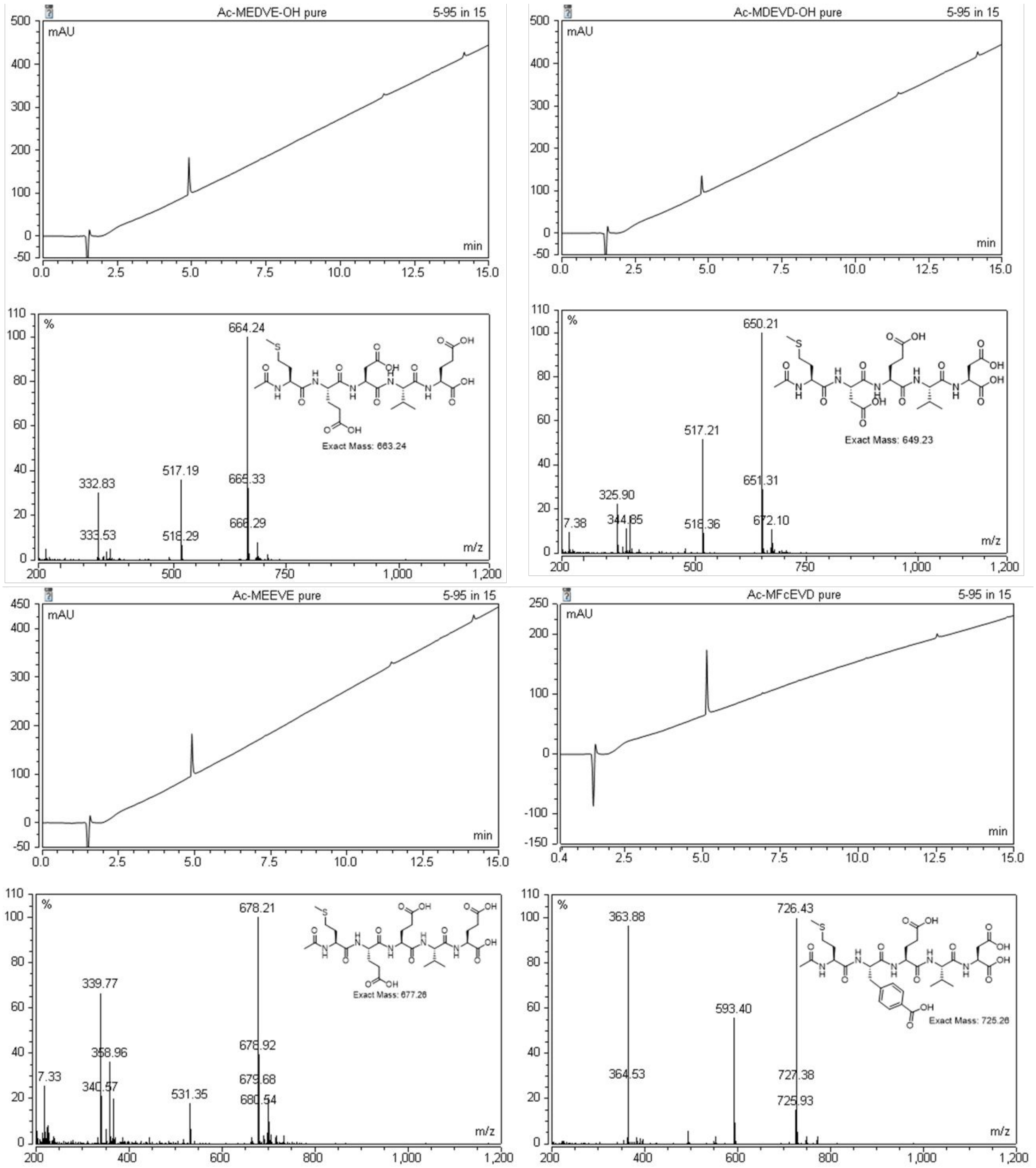

Figure S6. LC-MS analysis of peptides **5 – 8**. Upper panel: UV detector at 220nm. Lower panel: experimental mass in postive ion mode $[M+1H]^{1+}$

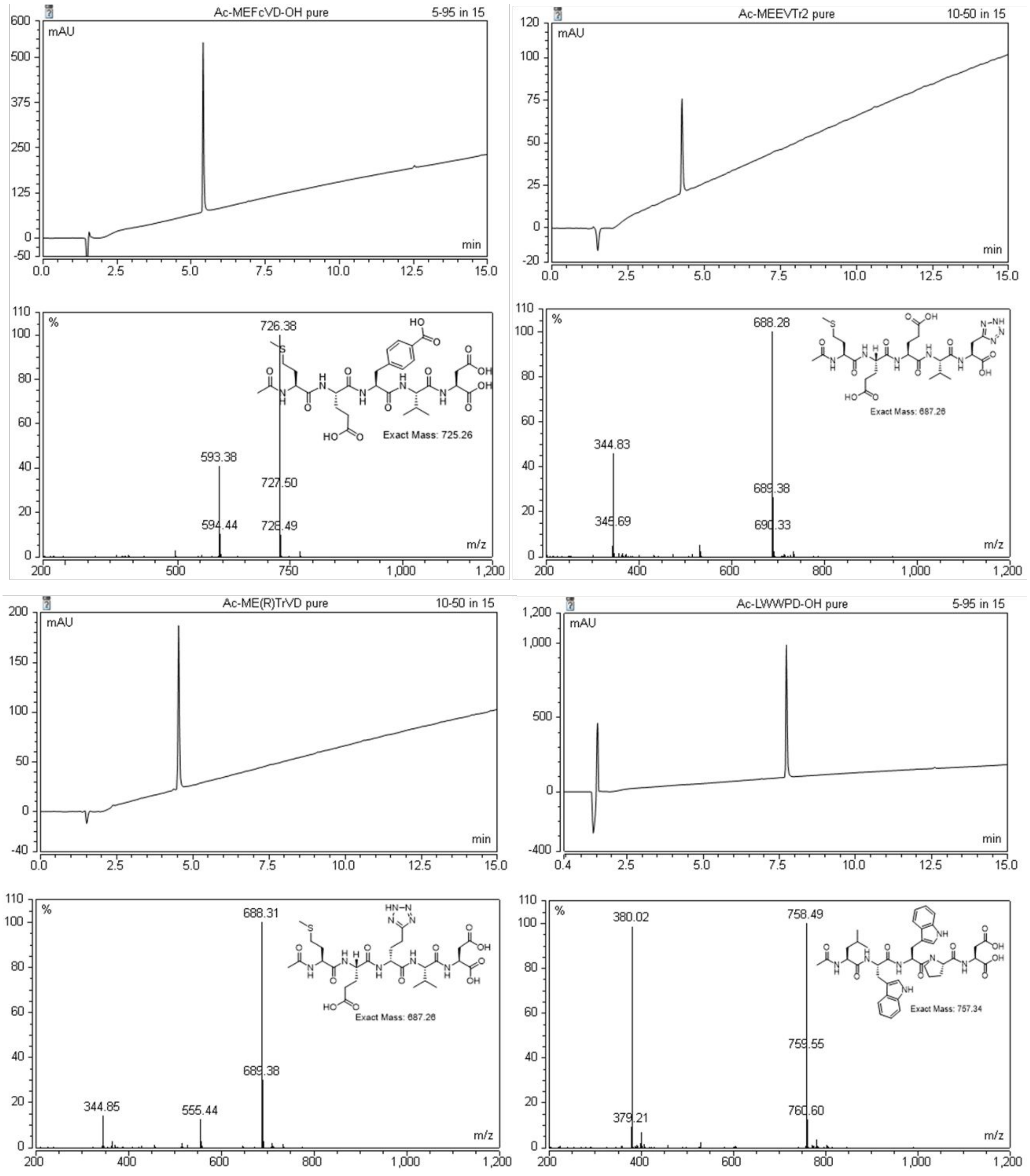

Figure S7. LC-MS analysis of peptides **9 – 12**. Upper panel: UV detector at 220nm. Lower panel: experimental mass in postive ion mode $[M+1H]^{1+}$

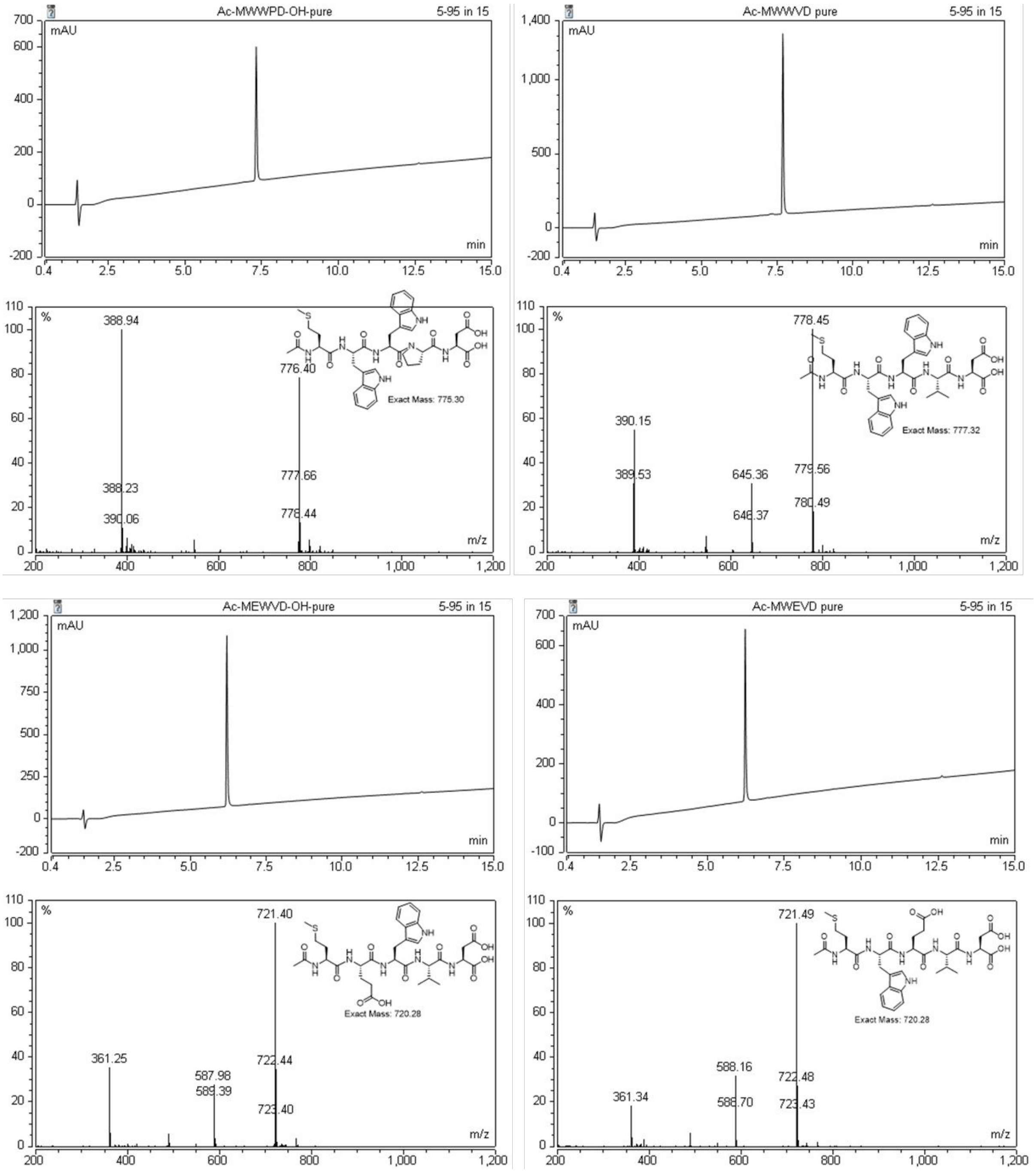

Figure S8. LC-MS analysis of peptides **13 – 16**. Upper panel: UV detector at 220nm. Lower panel: experimental mass in postive ion mode $[M+1H]^{1+}$

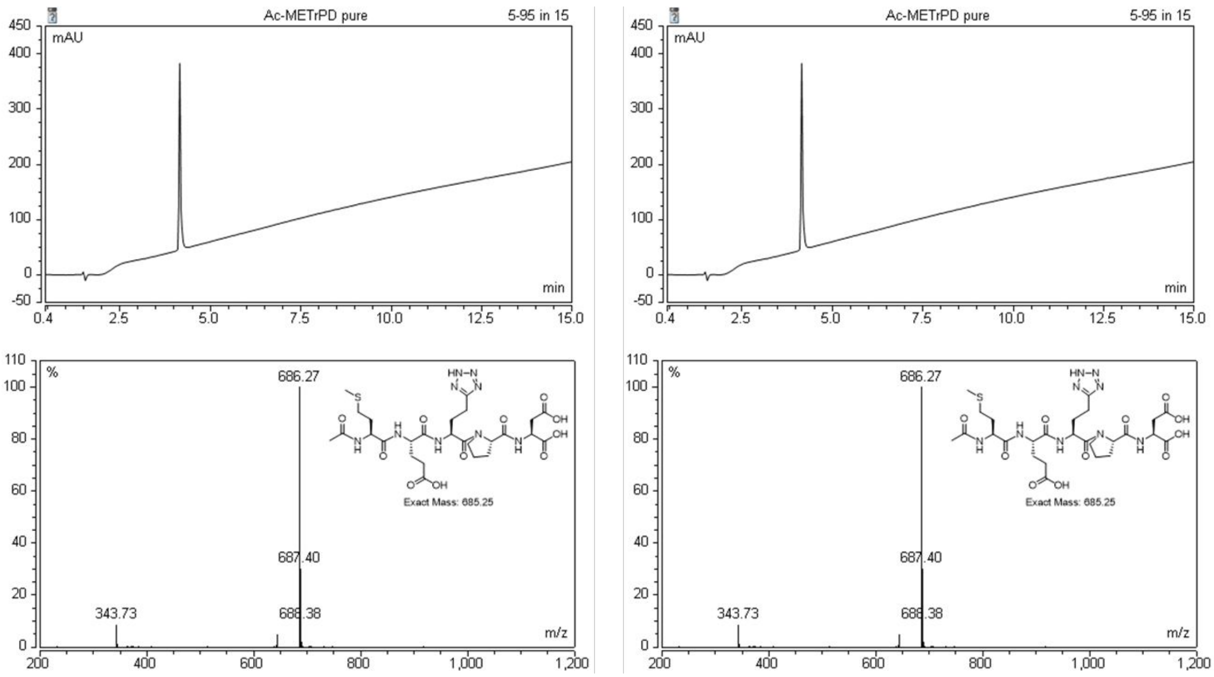

Figure S9. LC-MS analysis of peptides **17** and **18**. Upper panel: UV detector at 220nm. Lower panel: experimental mass in postive ion mode $[M+1H]^{1+}$

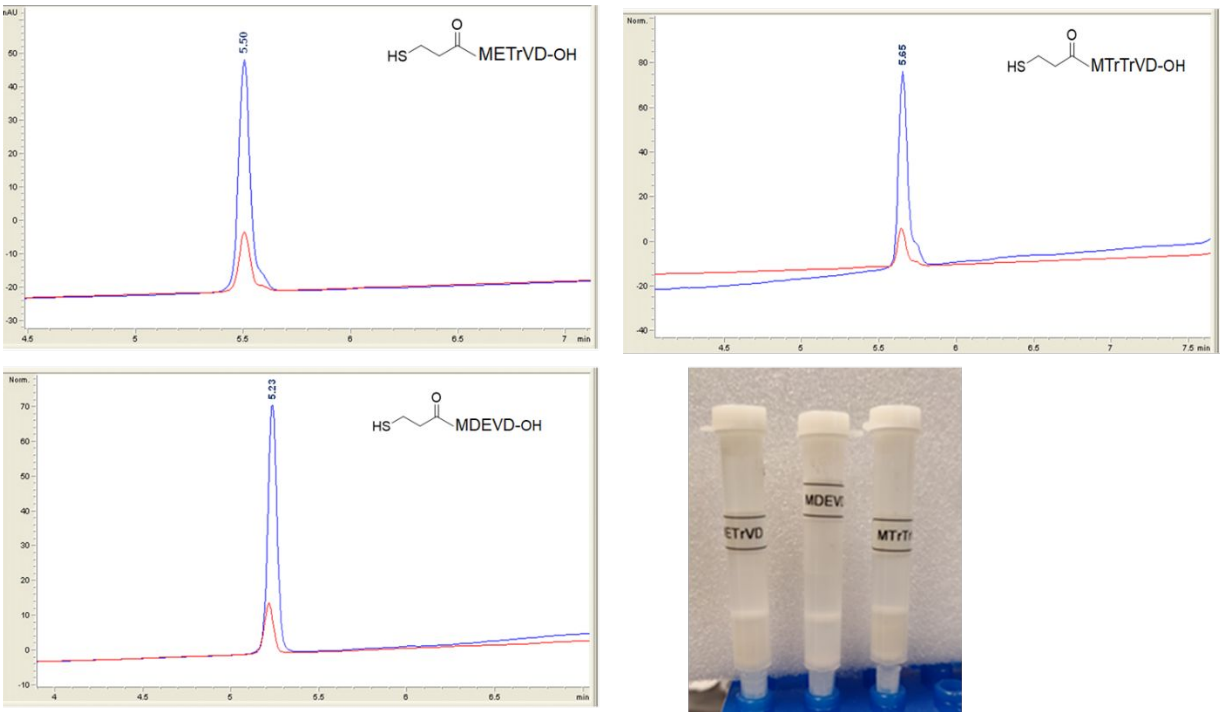

Figure S10. Immobilization of thiol-derivatized peptides to agarose (SulfoLink®). HPLC analysis of the coupling solution before (blue trace) and after (red trace) reaction. **IP-1, IP-2 and IP-5** were stored in PBS (pH=7.4) with 0.05% NaN_3

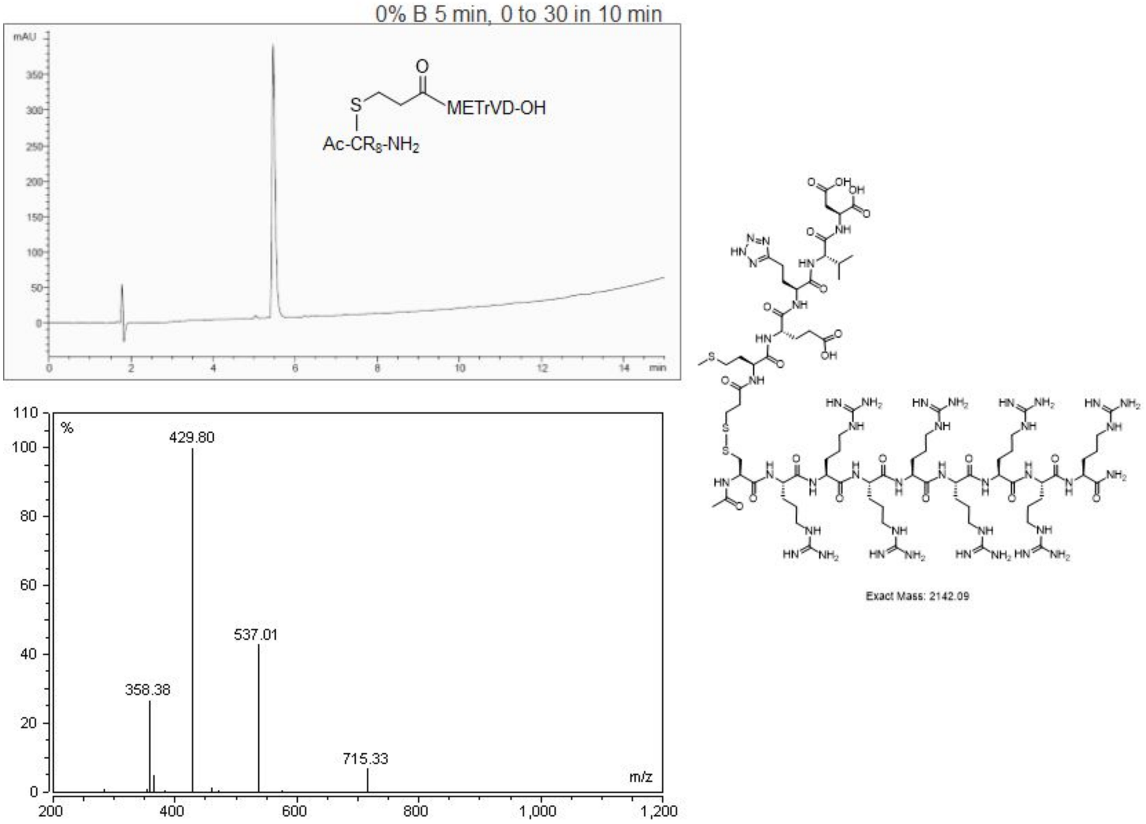

Figure S11. LC-MS analysis of **CPP-1.** Upper panel: UV detector at 220nm. Lower panel: experimental mass.

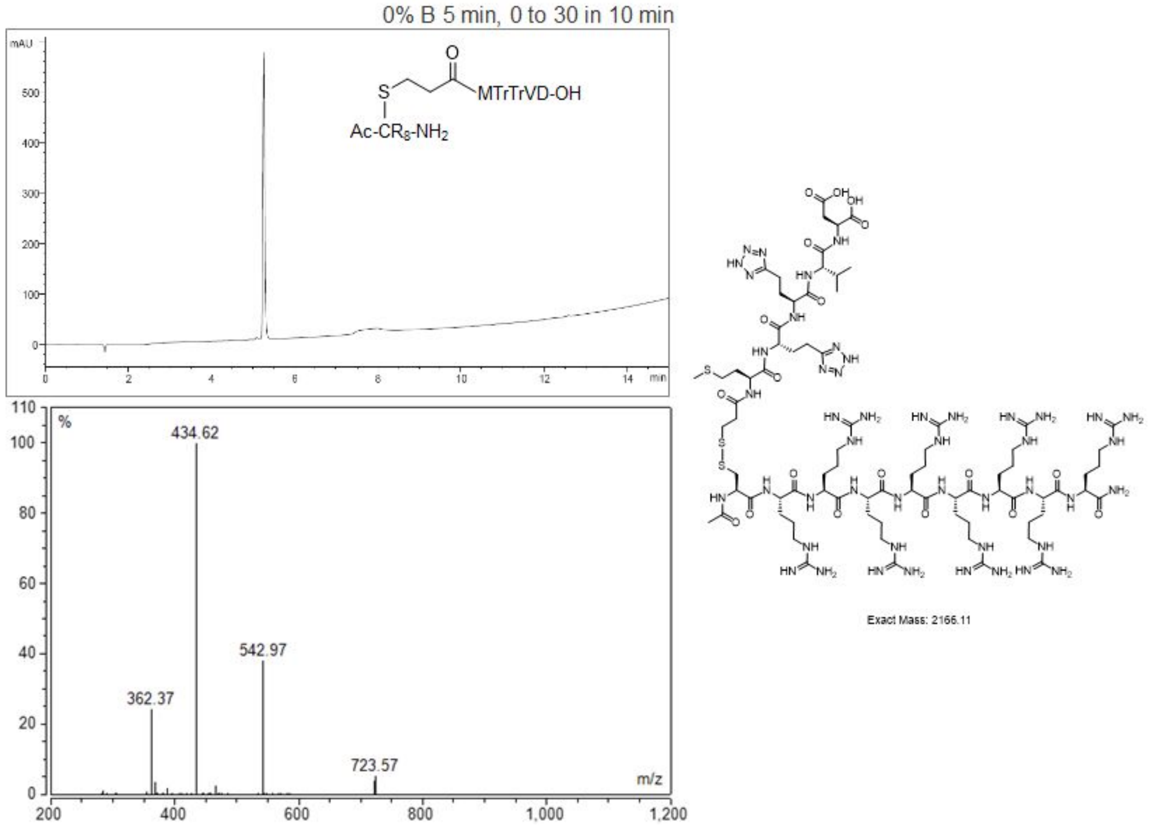

Figure S12. LC-MS analysis of **CPP-2.** Upper panel: UV detector at 220nm. Lower panel: experimental mass. ESIMS *m/z* 434.62 calcd. for 434.03 $C_{80}H_{151}N_{47}O_{19}S_3$ $[M+5H]^{5+}$

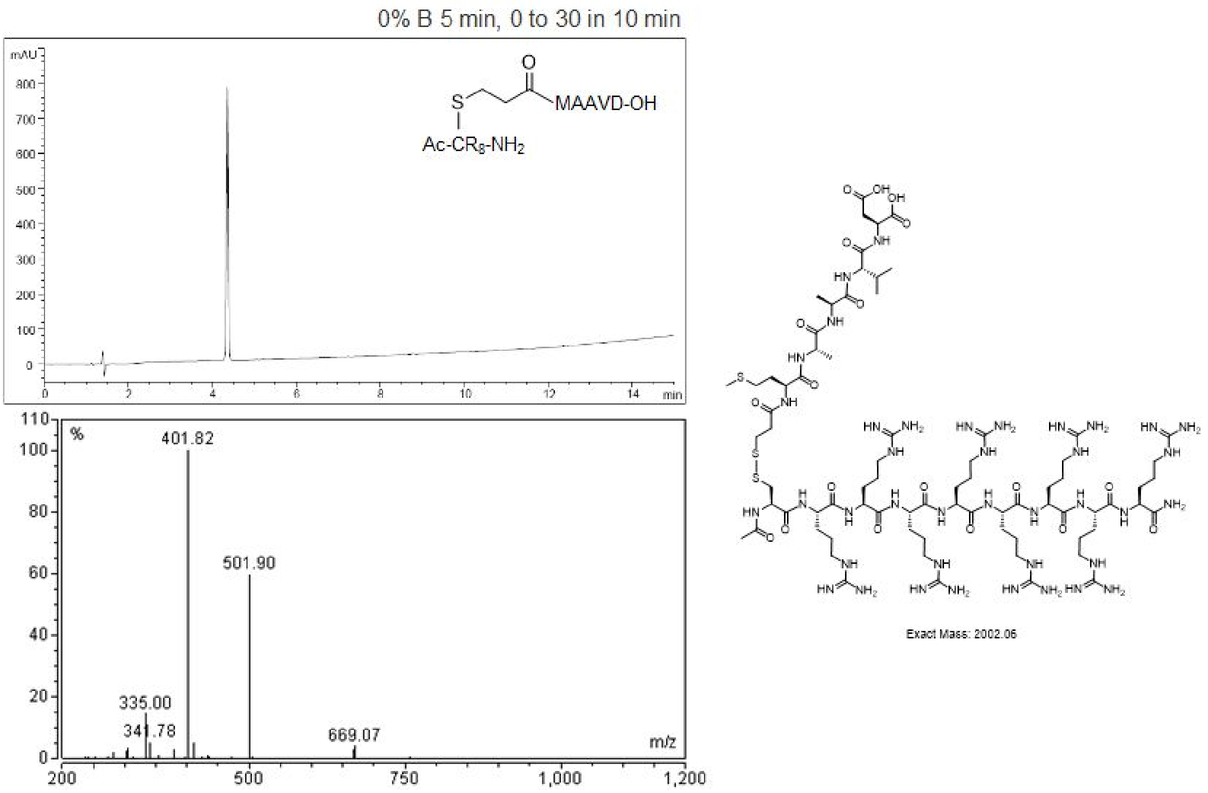

Figure S13. LC-MS analysis of **CPP-19.** Upper panel: UV detector at 220nm. Lower panel: experimental mass. ESIMS *m/z* 401.82 calcd. for 401.42 $C_{76}H_{148}N_{39}O_{19}S_3$ $[M+5H]^{5+}$
